# Food biodiversity and total and cause-specific mortality in 9 European countries: An analysis of a prospective cohort study

**DOI:** 10.1371/journal.pmed.1003834

**Published:** 2021-10-18

**Authors:** Giles T. Hanley-Cook, Inge Huybrechts, Carine Biessy, Roseline Remans, Gina Kennedy, Mélanie Deschasaux-Tanguy, Kris A. Murray, Mathilde Touvier, Guri Skeie, Emmanuelle Kesse-Guyot, Alemayehu Argaw, Corinne Casagrande, Geneviève Nicolas, Paolo Vineis, Christopher J. Millett, Elisabete Weiderpass, Pietro Ferrari, Christina C. Dahm, H. Bas Bueno-de-Mesquita, Torkjel M. Sandanger, Daniel B. Ibsen, Heinz Freisling, Stina Ramne, Franziska Jannasch, Yvonne T. van der Schouw, Matthias B. Schulze, Konstantinos K. Tsilidis, Anne Tjønneland, Eva Ardanaz, Stina Bodén, Lluís Cirera, Giuliana Gargano, Jytte Halkjær, Paula Jakszyn, Ingegerd Johansson, Verena Katzke, Giovanna Masala, Salvatore Panico, Miguel Rodriguez-Barranco, Carlotta Sacerdote, Bernard Srour, Rosario Tumino, Elio Riboli, Marc J. Gunter, Andrew D. Jones, Carl Lachat

**Affiliations:** 1 Department of Food Technology, Safety and Health, Faculty of Bioscience Engineering, Ghent University, Ghent, Belgium; 2 Nutritional Epidemiology Group, Nutrition and Metabolism Section, International Agency for Research on Cancer, World Health Organization, Lyon, France; 3 Nutritional Methodology and Biostatistics Group, Nutrition and Metabolism Section, International Agency for Research on Cancer, World Health Organization, Lyon, France; 4 Bioversity International, Heverlee, Belgium; 5 Department of Agrotechnology and Food Sciences, Wageningen University & Research, Wageningen, the Netherlands; 6 Global Alliance for Improved Nutrition (GAIN), Washington, DC, United States of America; 7 Sorbonne Paris Nord University, Inserm U1153, Inrae U1125, Cnam, Nutritional Epidemiology Research Team (EREN), Epidemiology and Statistics Research Center—University of Paris (CRESS), Bobigny, France; 8 MRC Centre for Global Infectious Disease Analysis, School of Public Health, Imperial College London, London, United Kingdom; 9 MRC Unit The Gambia at London School of Hygiene & Tropical Medicine, Fajara, Banjul, The Gambia; 10 Department of Community Medicine, Faculty of Health Sciences, UiT, The Arctic University of Norway, Tromsø, Norway; 11 Department of Population and Family Health, Institute of Health, Jimma University, Jimma, Ethiopia; 12 Biomarkers Group, Nutrition and Metabolism Section, International Agency for Research on Cancer, World Health Organization, Lyon, France; 13 Department of Epidemiology and Biostatistics, School of Public Health, Imperial College London, London, United Kingdom; 14 Public Health Policy Evaluation Unit, School of Public Health, Imperial College London, London, United Kingdom; 15 International Agency for Research on Cancer, World Health Organization, Lyon, France; 16 Department of Public Health, Aarhus University, Aarhus, Denmark; 17 Department for Determinants of Chronic Diseases (DCD), National Institute for Public Health and the Environment (RIVM), Bilthoven, the Netherlands; 18 Institute of Environmental Medicine, Karolinska Institute, Stockholm, Sweden; 19 Department of Clinical Sciences, Lund University, Malmö, Sweden; 20 Department of Molecular Epidemiology, German Institute of Human Nutrition Potsdam-Rehbruecke, Nuthetal, Germany; 21 German Center for Diabetes Research (DZD), München-Neuherberg, Germany; 22 NutriAct—Competence Cluster Nutrition Research Berlin-Potsdam, Nuthetal, Germany; 23 Julius Center for Health Sciences and Primary Care, University Medical Center Utrecht, Utrecht University, Utrecht, the Netherlands; 24 Institute of Nutritional Sciences, University of Potsdam, Nuthetal, Germany; 25 Department of Hygiene and Epidemiology, University of Ioannina School of Medicine, Ioannina, Greece; 26 Danish Cancer Society Research Center, Copenhagen, Denmark; 27 Department of Public Health, University of Copenhagen, Copenhagen, Denmark; 28 Navarra Public Health Institute, Pamplona, Spain; 29 IdiSNA, Navarra Institute for Health Research, Pamplona, Spain; 30 Biomedical Research Networking Center for Epidemiology and Public Health (CIBERESP), Madrid, Spain; 31 Department of Radiation Sciences, Oncology, Umeå University, Umeå, Sweden; 32 Department of Epidemiology, Murcia Regional Health Council—IMIB-Arrixaca, Murcia, Spain; 33 Department of Health and Social Sciences, University of Murcia, Murcia, Spain; 34 Epidemiology and Prevention Unit, Fondazione IRCCS Istituto Nazionale dei Tumori, Milan, Italy; 35 Unit of Nutrition and Cancer, Cancer Epidemiology Research Program, Catalan Institute of Oncology (ICO-IDIBELL), L’Hospitalet de Llobregat, Barcelona, Spain; 36 Blanquerna School of Health Sciences, Ramon Llull University, Barcelona, Spain; 37 School of Dentistry, Cariology, Department of Odontology, Umeå University, Umeå, Sweden; 38 Division of Cancer Epidemiology, German Cancer Research Center (DKFZ), Heidelberg, Germany; 39 Cancer Risk Factors and Life-Style Epidemiology Unit, Institute for Cancer Research, Prevention and Clinical Network (ISPRO), Florence, Italy; 40 Dipartimento di Medicina Clinica e Chirurgia, Federico II University, Naples, Italy; 41 Andalusian School of Public Health (EASP), Granada, Spain; 42 Instituto de Investigación Biosanitaria de Granada (ibs.GRANADA), Universidad de Granada, Granada, Spain; 43 Unit of Cancer Epidemiology, Città della Salute e della Scienza University-Hospital and Centre for Cancer Prevention (CPO), Turin, Italy; 44 Cancer Registry and Histopathology Department, Azienda Sanitaria Provinciale Ragusa (ASP 7), Ragusa, Italy; 45 Department of Nutritional Sciences, School of Public Health, University of Michigan, Ann Arbor, Michigan, United States of America; University of Cambridge, UNITED KINGDOM

## Abstract

**Background:**

Food biodiversity, encompassing the variety of plants, animals, and other organisms consumed as food and drink, has intrinsic potential to underpin diverse, nutritious diets and improve Earth system resilience. Dietary species richness (DSR), which is recommended as a crosscutting measure of food biodiversity, has been positively associated with the micronutrient adequacy of diets in women and young children in low- and middle-income countries (LMICs). However, the relationships between DSR and major health outcomes have yet to be assessed in any population.

**Methods and findings:**

We examined the associations between DSR and subsequent total and cause-specific mortality among 451,390 adults enrolled in the European Prospective Investigation into Cancer and Nutrition (EPIC) study (1992 to 2014, median follow-up: 17 years), free of cancer, diabetes, heart attack, or stroke at baseline. Usual dietary intakes were assessed at recruitment with country-specific dietary questionnaires (DQs). DSR of an individual’s yearly diet was calculated based on the absolute number of unique biological species in each (composite) food and drink. Associations were assessed by fitting multivariable-adjusted Cox proportional hazards regression models. In the EPIC cohort, 2 crops (common wheat and potato) and 2 animal species (cow and pig) accounted for approximately 45% of self-reported total dietary energy intake [median (*P*_10_–*P*_90_): 68 (40 to 83) species consumed per year]. Overall, higher DSR was inversely associated with all-cause mortality rate. Hazard ratios (HRs) and 95% confidence intervals (CIs) comparing total mortality in the second, third, fourth, and fifth (highest) quintiles (Qs) of DSR to the first (lowest) Q indicate significant inverse associations, after stratification by sex, age, and study center and adjustment for smoking status, educational level, marital status, physical activity, alcohol intake, and total energy intake, Mediterranean diet score, red and processed meat intake, and fiber intake [HR (95% CI): 0.91 (0.88 to 0.94), 0.80 (0.76 to 0.83), 0.69 (0.66 to 0.72), and 0.63 (0.59 to 0.66), respectively; *P*_*Wald*_ < 0.001 for trend]. Absolute death rates among participants in the highest and lowest fifth of DSR were 65.4 and 69.3 cases/10,000 person-years, respectively. Significant inverse associations were also observed between DSR and deaths due to cancer, heart disease, digestive disease, and respiratory disease. An important study limitation is that our findings were based on an observational cohort using self-reported dietary data obtained through single baseline food frequency questionnaires (FFQs); thus, exposure misclassification and residual confounding cannot be ruled out.

**Conclusions:**

In this large Pan-European cohort, higher DSR was inversely associated with total and cause-specific mortality, independent of sociodemographic, lifestyle, and other known dietary risk factors. Our findings support the potential of food (species) biodiversity as a guiding principle of sustainable dietary recommendations and food-based dietary guidelines.

## Abstract 2

Dr. Giles T. Hanley-Cook and colleagues, analyzed data from a prospective cohort study to investigate the association between food biodiversity, and total and cause-specific mortality in nine European countries.

## Author summary

**Why was this study done?:**

- Facilitating populations to transition to diets that are both nutritious and sustainable is a key challenge for human and environmental health. Dietary (between food group) diversity is increasingly advocated within the framework of dietary recommendations and food-based dietary guidelines.
- Food biodiversity, defined as the variety of plants, animals, and other organisms that are consumed between and within food groups globally, is a potential lever for improved public and planetary health. Nevertheless, scientific evidence is a prerequisite to understand the associations between metrics of food biodiversity and major health outcomes.
- Thus far, evidence regarding the positive association between dietary species richness (DSR), i.e., the absolute number of unique species consumed by an individual, and micronutrient adequacy of diets is limited to low- and middle-income countries (LMICs).

**What did the researchers do and find?:**

- This study assessed the relationships between DSR and all-cause and cause-specific mortality in the large and diverse European population that constitutes the European Prospective Investigation into Cancer and Nutrition (EPIC) cohort, including 451,390 adults from 9 European countries with 46,627 recorded deaths between 1992 and 2014. In addition, this study provides a picture of the usual consumption of food and beverage species across 9 European countries.
- Higher DSR was inversely associated with total mortality rate and cause-specific deaths due to cancer, heart disease, respiratory disease, and digestive disease, independent of other established components of diet quality. Overall, self-reported total energy intake was derived from a narrow range of species.

**What do these findings mean?:**

- Our findings add to the evidence base on the relevance of food biodiversity, both between and within food groups, as a basis for public health strategies championing dietary (species) diversity in European countries.
- These findings will play a key role in communications about the merits of biodiversity stewardship, in the context of ongoing European/global debate on nutritious and sustainable diets for a stable Earth system.

## Introduction

Diets inextricably link human and environmental health [1]. The global food system is the primary driver of unprecedented Earth system biodiversity loss (e.g., monocropping, land degradation, and deforestation for agriculture) [2,3], while low-quality, nondiverse diets are responsible for the greatest burden of disease worldwide, affecting countries and populations at all levels of socioeconomic development [4]. The short- and long-term consequences of accelerated biodiversity collapse [5,6] and the triple burden of malnutrition [7,8] restrain sustainable and inclusive global development and convey unacceptable human consequences [9,10].

At present, rapid socioeconomic, demographic, and technological transitions, coupled with agricultural policies skewed toward a narrow range of staple crops, crop varieties, and animal species [11], are driving a progressive homogeneity of human diets [12,13]. In parallel, the associated global food systems, which are mainly focused on cheap calories, rather than nutrients, are redirected toward more resource-intensive, energy-dense, and nutrient-poor food species [14]. The Sustainable Development Goals (SDGs), the United Nations Decade of Action on Nutrition 2016–2025, and the Convention on Biological Diversity’s forthcoming post-2020 biodiversity agenda provide global and national stimuli to fast-track transition from business-as-usual to win-win scenarios for human and environmental health in the Anthropocene epoch [15].

Food biodiversity, defined as the variety of plants, animals, and other organisms (e.g., fungi and yeast cultures) that are used for food and drink, both cultivated and from the wild, has intrinsic potential to underpin diverse, nutritious diets and conserve (neglected and underutilized) finite genetic resources (i.e., biodiversity) [16,17]. Thus, the concept of food biodiversity potentially offers a unique and novel entry point to guide the development of sustainable food-based dietary guidelines (and interventions) cutting across human and planetary health [18,19]. Dietary diversity, which is conventionally measured as consumption between, rather than within nutrient-dense food groups, is a widely acknowledged and established public health recommendation to promote healthy, nutritionally adequate diets [20]. Furthermore, diets based on a wide diversity of (locally available, nonthreatened) biological species exert lower pressure on single species, hence increasing Earth system stability, resilience, ecosystem services, and enhanced productivity of natural and agricultural systems [5,21]. Observational studies have indicated consistently positive, but small associations between agricultural biodiversity [22] and forest patterns [23] with dietary diversity in low- and middle-income countries (LMICs). Nevertheless, environmental sustainability criteria, including biodiversity preservation in, e.g., Brazil, the Netherlands, and Sweden, are only explicitly included in 8 (quasi) official sustainable food-based dietary guidelines worldwide [24]. Moreover, globally, the potential dual benefits of at-scale adoption of national food-based dietary guidelines on human health and environmental impacts can be substantially improved [25].

Previous cross-sectional research indicated that higher dietary species richness (DSR), a recommended measure of food biodiversity, which captures both inter- and intra-food group diversity, was associated with increased micronutrient adequacy of diets in approximately 2,200 women of reproductive age and approximately 4,000 children aged 6 to 23 months in LMICs [26]. Furthermore, several prospective studies have reported lower mortality and chronic disease rates among participants with higher between food group diversity [27,28], within specific food group richness (e.g., fruit and vegetables) [29], and food item variety [30]. To date, however, the evidence base for relationships between food (species) biodiversity of whole dietary patterns and major human health outcomes is missing. In this study, we address the knowledge gap by assessing associations between DSR and total and cause-specific mortality in a large and diverse Pan-European cohort.

## Methods

Our research was reported using the STrengthening the Reporting of OBservational Studies in Epidemiology (STROBE)-nut checklist [31].

### Study population: The EPIC cohort

The European Prospective Investigation into Cancer and Nutrition (EPIC) study (http://epic.iarc.fr/) is an ongoing multicenter, prospective cohort study investigating metabolic, dietary, lifestyle, and environmental factors in relation to cancer and other chronic diseases. Between 1992 and 2000, more than 500,000 volunteers (25 to 70 years) were recruited from 10 European countries (23 administrative centers): Denmark, France, Germany, Greece, Italy, the Netherlands, Norway, Spain, Sweden, and the United Kingdom. Most of the participants were recruited from the general population residing in a given geographic area, town, or province. Exceptions were the cohorts of France (female members of a health insurance scheme for school employees), Utrecht (breast cancer screening attendees), Ragusa (blood donors and their spouses), and Oxford (mainly vegetarian and healthy eaters). All participants gave written informed consent and completed questionnaires on their diet, lifestyle, and medical history. The study was approved by the local ethics committees and by the Internal Review Board of the International Agency for Research on Cancer. Details of the study design, recruitment, and data collection have been previously published [32–34].

Of the 521,324 participants enrolled, 451,390 were included in the analyses, with 46,627 recorded deaths between 1992 and 2014. We excluded participants with missing lifestyle or dietary information, those with an extreme ratio of energy intake to energy requirement (top and bottom 1%, as these values were considered physiologically implausible [34]), volunteers with null follow-up, those with prevalent disease at baseline (history of cancer, cardiovascular diseases [CVDs], and diabetes), and all participants from the EPIC-Greece cohort, due to administrative constraints (S1 Fig).

### Baseline data collection

An extensive and standardized phenotypic characterization was performed for each participant upon enrollment. Questionnaires were used to collect sociodemographic information, educational level (standardized for the whole cohort), personal and familial history of diseases, lifestyle (e.g., smoking, alcohol use, and physical activity), and menstrual and reproductive history of women. Anthropometric measurements (e.g., height, weight, waist, and hip circumferences) were performed in all centers (except France, Oxford, and Norway: self-reported data) [35]. Height and weight were complemented with available self-reported values or imputed with center-, age-, and gender-specific average values when missing. Body mass index (BMI) was calculated as weight divided by height squared (kg/m^2^).

### Dietary intake assessment

Usual dietary intake was assessed for each individual at recruitment using country- or center-specific validated dietary questionnaires (DQs) developed to capture the geographical specificity of an individual’s diet over the preceding year. The type of DQ used differed according to study centers and included self- or interviewer-administered semiquantitative food frequency questionnaires (FFQs) with an estimation of individual average portions or with the same standard portion assigned to all participants or diet history questionnaires combining an FFQ and 7-day dietary records. In most centers, DQs were self-administered, with the exception of Ragusa, Naples, and Spain, where face-to-face interviews were conducted. Extensive quantitative DQs were used in northern Italy, the Netherlands, and Germany, which were structured by meals in Spain, France, and Ragusa. Semiquantitative FFQs were used in Denmark, Norway, Naples, Umeå, and the UK, while an FFQ was combined with a 7-day record on hot meals in Malmö [33]. Post-harmonization of DQ data was conducted, following standardized procedures (e.g., decomposing recipes into ingredients), to obtain a standardized food list for which the level of detail is comparable between countries. The EPIC food composition database comprises more than 11,000 food and beverage items reflecting the specificities of each country [36].

### Food biodiversity computation

**Fig 1:**
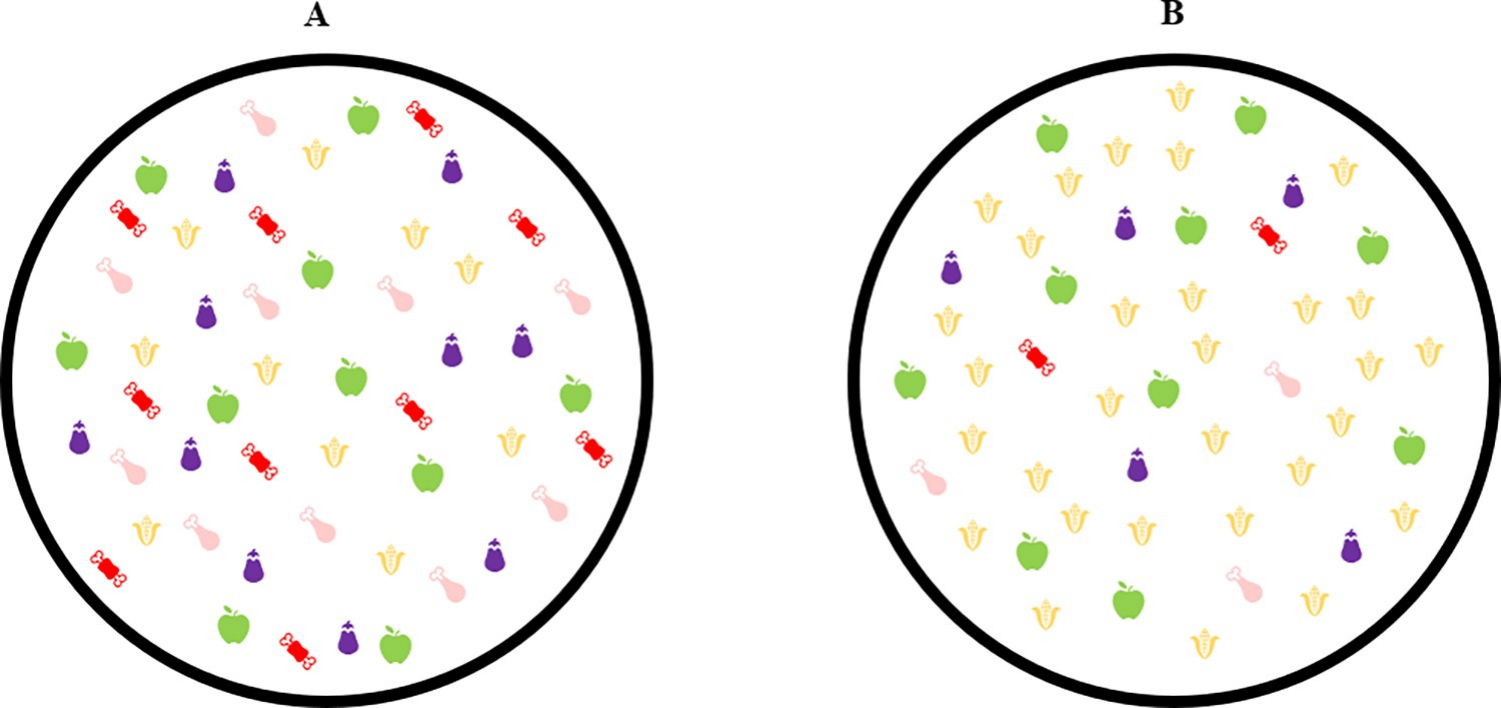
Partitioning food biodiversity in 2 dietary patterns, which both consist of 50 food and drink items. Distinct species are indicated by their color. Richness is the absolute number of species: In both dietary patterns, it is equal to 5. Evenness is the equitability of the species abundance distribution in the diet: In dietary pattern A, all species are present in an equal abundance (e.g., frequency) and so it is perfectly even, while dietary pattern B is very uneven since it is dominated by the yellow [*Zea mays* (maize)] species. Disparity is the level of similarity between species: For example, red [*Bos taurus* (cow)] and pink [*Gallus gallus* (chicken)] species are more similar to each other, e.g., nutritionally and taxonomically, than the purple [*Solanum melongena* (aubergine)] species.

(Bio)diversity can be partitioned into 3 components: richness, evenness, and disparity (Fig 1) [37]. Nevertheless, our study focuses only on DSR, previously recommended as the most appropriate measure of food biodiversity for dietary intake studies, as we aim to inform food-based interventions and policy based on a simple, crosscutting indicator of human and planetary health [26]. We argue that species evenness, which is defined as a perfectly equal distribution of food and drinks in the diet, is neither desired from a nutritional [38] nor environmental protection perspective [39]. Hence, dietary evenness requires an arbitrary a priori selection of a relative abundance unit (e.g., energy, nutrients, weight, volume, and frequency) and “healthfulness” weighting factors [40,41]. Moreover, unlike for species richness, there is currently no consensus on the measurement of species evenness (e.g., Shannon entropy, Berry–Simpson, and Pielou’s index) [37]. Dietary disparity is defined for our research purposes as the consumption of foods with distinct human health [42] and ecosystem attributes [43], rather than the more narrow, but well-established measures of nutritional food group diversity [44]. Similarly to evenness, ecological metrics of species dissimilarity are based on an inconsistent selection (and number) of phylogenetic, functional, and/or morphological traits (e.g., Rao’s quadratic diversity and Jaccard index) [37].

Therefore, for the 451,390 participants included in our analysis, food biodiversity in an individual’s diet was calculated based on the absolute number of unique biological species in each (composite) food, drink, and recipe, using the European Food Safety Authority’s FoodEx2 food classification and description system [45] in combination with the detailed EPIC food classification system (NCLASS). Food items consumed “never or less than once per month” (on average) were recalled under one category; accordingly, these species did not count toward DSR. Moreover, quantities (g/day) were disregarded for overall DSR computation, since our interest is the sum of distinct species consumed per year (i.e., DQs recalled dietary intake over the preceding 12 months). However, relative quantities were considered during sensitivity analyses, which excluded species consumed in trivial amounts (see below). Furthermore, although a species can be consumed multiple times per year, potentially from diverse functional food groups (e.g., chicken meat and eggs, which are nutritionally disparate), through a “biodiversity conservation” lens, it contributes only one species to an individual’s DSR in all scenarios (taxonomically identical: *Gallus gallus*). Here, we consider that high food biodiversity is thus a combination of multiple species that act synergistically and complimentary for both human and environmental health (e.g., ecological and net nutritional benefits from the Mesoamerican combination of corn, beans, and squash, known as the “three sisters”) [21,46].

For all countries, composite dishes were decomposed into their ingredients (species) using standard recipes. Therefore, herbs and spices and other ingredients potentially used in small amounts, for which we cannot be certain if they were added to the recipe by each EPIC participant, might bias/inflate the true value of an individual’s DSR. To assess the impact of food and drink species consumed in relatively small quantities, we calculated 3 different scenarios of DSR, namely the following:

- overall DSR, including all foods consumed in the EPIC food list (thus, also ingredients derived from standard recipes regardless of quantities);
- DSR excluding the lowest 5% species intake (g/day) from each EPIC food group (group/subgroup specific); and
- DSR excluding the lowest 10% species intake (g/day) from each EPIC food group (group/subgroup specific).

### Follow-up for vital status and cause of death

Data on vital status and cause and date of death were obtained using record linkages with population-based cancer registries, boards of health, health insurance registries, pathology registries and mortality registries (Denmark, Italy, the Netherlands, Norway, Spain, Sweden, and the UK), or through active follow-up and next of kin (France and Germany). Germany identified deceased individuals from undelivered follow-up mailings and subsequent enquiries to municipality registries, regional health department, physicians, or hospitals. In France, information on deceased participants was obtained using the database of health insurance for school employees and national death index. The end of follow-up/closure dates of the study period varied between 2009 and 2014 depending on the countries.

Cause-specific mortality data were coded according to the 10th revision of the International Statistical Classification of Diseases, Injuries, and Causes of Death (ICD-10) [47]. Causes of death assessed include heart disease [i.e., coronary heart disease (CHD) (ICD-10 codes: I20 to I25) and CVD other than CHD (I00 to I99, excluding I20 to I25)], cancer (C00 to D48), diseases of the respiratory system (J00 to J99), and diseases of the digestive system (K00 to K93). Total mortality (main outcome) was defined as mortality from all causes, except external causes of death (S00 to T98 and V01 to Y98).

### Statistical analyses

Statistical analyses were preplanned and followed the plan detailed in the project proposal that was submitted to and approved (March 2019) by the EPIC Steering Committee (see S1 Text). Unadjusted absolute death rates were calculated as the number of cases per 10,000 person-years in Q_5_ and Q_1_ of DSR, respectively. Associations between food biodiversity [DSR per year; count variable and quintiles (Qs)] and total and cause-specific mortality were characterized [hazard ratio (HR) and 95% confidence interval (CI)] using multivariable-adjusted Cox proportional hazard regression models with age as the primary underlying time variable. The Breslow method was adopted for handling ties. Examination of the Schoenfeld residuals, according to follow-up time (years) for Qs of DSR, confirmed that the assumptions of proportionality were satisfied. Overall survival curves by Q of DSR were generated using the Kaplan–Meier method (S2 Fig). Participants contributed person-time to the model until their date of death, their date of emigration/loss to follow-up, or end-of-follow-up, whichever occurred first. *P*-values for linear trend were calculated with the use of the Wald test of a pseudo-continuous score variable, based on the median number of species consumed per year for each Q of DSR. Nonlinear associations between DSR and total mortality were examined nonparametrically with restricted cubic splines [48]. *P*-value for nonlinear trend was calculated with the use of the likelihood ratio test, comparing the model with only the linear term to the model with the linear and the cubic spline terms.

Pooled cohort models were stratified by sex, age at recruitment (1-year intervals), and study center (“strata” option in proc phreg, SAS) and multivariable-adjusted for confounding factors using a 5% change-in-estimate criterion for *β*-coefficients (applied to all variables reported in Table 1, due to limited knowledge on factors relating to DSR): smoking status (current, 1 to 15 cigarettes/day; current, 16 to 25 cigarettes/day; current, 26+ cigarettes/day; current, pipe/cigar/occasional; current/former, missing; former, quit 11 to 20 years; former, quit 20+ years; former, quit ≤10 years; never; unknown), educational level, as a proxy variable for socioeconomic status [longer education (including university degree, technical, or professional school); secondary school; primary school completed; not specified], marital status (single, divorced, separated, or widowed; married or living together; unknown), physical activity (Cambridge index: active; moderately active; moderately inactive; inactive; missing), alcohol intake at recruitment (g/day), total energy intake (kcal/day), the 18-point relative Mediterranean diet score, as an indicator for an overall healthy diet [49], the consumption of red and processed meat (g/day) [50], and fiber intake (g/day; i.e., to reflect carbohydrate quality [51], such as whole grains). Possible multicollinearity of (dietary) variables included in our models were assessed by “collin” and “vif” options in proc phreg, SAS (all condition indices <30 and variance inflation factors <3, respectively). When data on categorical covariates were missing, a “missing class” was introduced to the model.

**Table 1:** Baseline characteristics of participants overall and by Qs of food biodiversity, EPIC cohort, 1992 to 2014.

			Qs of DSR				
	*N*	All	Q_1_ (*n* = 93,179)	Q_2_ (*n* = 96,994)	Q_3_ (*n* = 90,983)	Q_4_ (*n* = 90,424)	Q_5_ (*n* = 79,810)
		*N* (%) Mean ± SD	*N* (%) Mean ± SD	*N* (%) Mean ± SD	*N* (%) Mean ± SD	*N* (%) Mean ± SD	*N* (%) Mean ± SD
**DSR, species per year** ^**a**^	451,390	68 (40 to 83)	41 (31 to 47)	58 (50 to 64)	69 (65 to 72)	77 (73 to 81)	83 (82 to 89)
**Age at recruitment, years**	451,390	51 ± 10	52 ± 8	51 ± 9	49 ± 11	52 ± 9	51 ± 11
**Sex**	451,390						
Male		131,782 (29.2)	20,488 (22)	27,100 (27.9)	26,042 (28.6)	28,936 (32)	29,216 (36.6)
Female		319,608 (70.8)	72,691 (78)	69,894 (72.1)	64,941 (71.4)	61,488 (68)	50,594 (63.4)
**Country**	451,390						
Denmark		55,014 (12.2)	947 (1.0)	8,291 (80.5)	16,642 (18.3)	28,106 (31.1)	1,028 (1.3)
France		67,920 (15)	17,403 (18.7)	14,085 (14.5)	15,888 (17.5)	15,722 (17.4)	4,822 (6)
Germany		49,352 (10.9)	98 (0.1)	1,495 (1.5)	4,182 (4.6)	15,678 (17.3)	27,899 (35)
Italy		44,547 (9.9)	923 (1)	12,879 (13.3)	15,770 (17.3)	11,844 (13.1)	3,131 (3.9)
the Netherlands		36,538 (8.1)	926 (1)	14,165 (14.6)	17,531 (19.3)	3,916 (4.3)	0 (0)
Norway		33,967 (7.5)	25,488 (27.4)	8,479 (8.7)	0 (0)	0 (0)	0 (0)
Spain		39,990 (8.9)	31,535 (33.8)	8,359 (8.6)	92 (0.1)	4 (0)	0 (0)
Sweden		48,690 (10.8)	15,721 (16.9)	28,997 (29.9)	3,966 (4.4)	6 (0)	0 (0)
UK		75,372 (16.7)	138 (0.1)	244 (0.3)	16,912 (18.6)	15,148 (16.8)	42,930 (53.8)
**Marital status**	451,390						
Single, divorced, separated or widowed		72,765 (16.1)	9,676 (10.4)	15,669 (16.2)	18,008 (19.8)	12,982 (14.4)	16,430 (20.6)
Married or living together		270,976 (60.0)	45,210 (48.5)	62,090 (64.0)	54,780 (60.2)	48,008 (53.1)	60,888 (76.3)
Unknown		107,649 (23.8)	38,293 (41.1)	19,235 (19.8)	18,195 (20.0)	29,434 (32.6)	2,492 (3.1)
**Educational level**	451,390						
None or primary school completed		126,948 (28.1)	41,011 (44)	32,422 (33.4)	19,852 (21.8)	20,603 (22.8)	13,060 (16.4)
Technical/professional school		104,016 (23)	16,096 (17.3)	20,926 (21.6)	20,765 (22.8)	23,121 (25.6)	23,108 (29)
Secondary school		94,181 (20.9)	19,187 (20.6)	23,608 (24.3)	22,782 (25)	18,370 (20.3)	10,234 (12.8)
Longer education (including university degree)		109,362 (24.2)	15,694 (16.8)	19,057 (19.6)	24,660 (27.1)	24,804 (27.4)	25,147 (31.5)
Missing		16,883 (3.7)	1,191 (1.3)	981 (1)	2,924 (3.2)	3,526 (3.9)	8,261 (10.4)
**Smoking status**	451,390						
Never		219,854 (48.7)	44,424 (47.7)	47,361 (48.8)	45,196 (49.7)	43,014 (47.6)	39,859 (49.9)
Current		100,053 (22.2)	24,033 (25.8)	24,392 (25.1)	20,008 (22.0)	19,309 (21.4)	12,311 (15.4)
Former		123,034 (27.3)	21,813 (23.4)	23,785 (24.5)	24,693 (27.1)	26,848 (29.7)	25,895 (32.4)
Unknown		8,449 (1.9)	2,909 (3.1)	1,456 (1.5)	1,086 (1.2)	1,253 (1.4)	1,745 (2.2)
**Physical activity (Cambridge index)**	451,390						
Inactive		88,276 (19.6)	22,188 (23.8)	20,635 (21.3)	14,439 (15.9)	13,570 (15)	17,444 (21.9)
Moderately inactive		150,393 (33.3)	29,545 (31.7)	31,027 (32)	30,253 (33.3)	31,377 (34.7)	28,191 (35.3)
Moderately active		120,554 (26.7)	28,694 (30.8)	25,870 (26.7)	22,950 (25.2)	23,741 (26.3)	19,299 (24.2)
Active		83,346 (18.5)	10,956 (11.8)	17,212 (17.7)	20,847 (22.9)	20,828 (23)	13,503 (16.9)
Missing		8,821 (2)	1,796 (1.9)	2,250 (2.3)	2,494 (2.7)	908 (1)	1,373 (1.7)
**BMI** ^**b**^ **, kg/m** ^**2**^	451,390	25.3 ± 4.2	25.8 ± 4.5	25.3 ± 4.2	24·7 ± 4	25.2 ± 4.1	25.4 ± 4.1
**Height** ^**b**^ **, cm**	451,390	166 ± 9	164 ± 8	166 ± 9	167 ± 9	167 ± 9	167 ± 9
**Weight** ^**b**^ **, kg**	451,390	70.0 ± 13.6	69.6 ± 13.1	69.9 ± 13.6	68.8 ± 13·6	70.4 ± 14.0	71.3 ± 13.8
**Family history of breast cancer, yes** ^**c**^	144,611	12,451 (8.6)	4,036 (7.4)	3,430 (10.3)	2,457 (10.2)	1,564 (8.6)	964 (6.8)
**Family history of colorectal cancer, yes** ^**c**^	115,617	9,785 (8.5)	3,324 (7.6)	2,021 (104)	1,303 (10.2)	1,493 (9)	1,644 (7.2)
**Dietary intake** ^**a**^	451,390						
Energy intake, kcal/day		1,999 (1,352 to 2,904)	1,833 (1,232 to 2,756)	1,962 (1,324 to 2,861)	2,033 (1,402 to 2,909)	2,124 (1,461 to 3,023)	2,043 (1,409 to 2,944)
Potatoes and other tuber intake, g/day		78 (20 to 184)	72 (21 to 182)	86 (17 to 213)	71 (16 to 182)	80 (21 to 180)	81 (29 to 151)
Vegetables intake, g/day		167 (68 to 364)	163 (61 to 379)	144 (46 to 329)	170 (74 to 370)	182 (84 to 364)	180 (84 to 371)
Legume intake, g/day		5 (0 to 42)	0 (0 to 66)	2 (0 to 40)	6.5 (0 to 42)	3 (0 to 31)	7 (1 to 35)
Fruit intake, g/day		193 (54 to 449)	184 (36 to 454)	194 (49 to 449)	209 (59 to 469)	197 (63 to 446)	181 (64 to 423)
Dairy product intake, g/day		285 (80 to 634)	256 (75 to 566)	286 (83 to 662)	281 (65 to 658)	284 (83 to 648)	322 (103 to 629)
Cereal intake, g/day		200 (102 to 359)	185 (90 to 319)	197 (100 to 359)	215 (114 to 387)	212 (112 to 375)	193 (97 to 351)
Meat intake, g/day		93 (26 to 177)	87 (33 to 174)	92 (40 to 173)	92 (2 to 175)	105 (28 to 189)	91 (32 to 170)
Red and processed meat intake, g/day		69 (15 to 142)	62 (20 to 132)	67 (25 to 139)	69 (2 to 144)	80 (17 to 152)	66 (18 to 138)
Fish and shellfish intake, g/day		29 (4 to 82)	47 (11 to 113)	23 (3 to 89)	17 (0 to 54)	32 (9 to 72)	28 (8 to 69)
Egg intake, g/day		14 (2 to 39)	16 (2 to 44)	12 (1 to 36)	14 (2 to 37)	16 (4 to 42)	10 (3 to 29)
Fat intake, g/day		24 (9 to 48)	23 (8 to 48)	27 (10 to 53)	23 (8 to 47)	23 (8 to 45)	24 (9 to 48)
Sugar and confectionery intake, g/day		32 (7 to 86)	23 (2 to 68)	29 (6 to 80)	35 (9 to 92)	39 (11 to 103)	35 (10 to 89)
Cakes and biscuits intake, g/day		31 (4 to 92)	26 (0 to 80)	31 (3 to 84)	27 (5 to 80)	28 (6 to 89)	45 (12 to 126)
Nonalcoholic beverage intake, g/day		1,046 (160 to 2,098)	556 (68 to 1,746)	833 (130 to 1,950)	1,153 (176 to 2,189)	1,326 (274 to 2,412)	1,192 (579 to 1,963)
Alcoholic beverage intake, g/day		75 (0 to 444)	43 (0 to 375)	56 (0 to 385)	83 (3 to 456)	119 (7 to 528)	94 (5 to 485)
Condiment and sauce intake, g/day		16 (3 to 46)	11 (1 to 41)	10 (1 to 37)	18 (4 to 48)	18 (6 to 44)	24 (8 to 56)
Soups and bouillon intake, g/day		22 (0 to 144)	7 (0 to 164)	17 (0 to 143)	27 (0 to 168)	19 (0 to 149)	28 (4 to 108)
Miscellaneous food intake, g/day		0 (0 to 14)	0 (0 to 7)	0 (0 to 7)	0 (0 to 38)	0 (0 to 11)	2 (0 to 15)
Fiber intake, g/day		22 (14 to 33)	20 (13 to 31)	21 (13 to 32)	23 (15 to 34)	23 (15 to 34)	22 (14 to 33)
**Mediterranean diet score, 0 to 18 points** ^**a**^	451,390	8 (4 to 12)	9 (5 to 13)	7 (4 to 12)	8 (4 to 13)	9 (5 to 12)	9 (5 to 12)
**Alcohol intake, categorical**	451,390						
Nondrinker		57,565 (12.8)	27,062 (29.0)	16,079 (16.6)	6,283 (6.9)	3,777 (4.2)	4,364 (5.5)
>0 to 6 g/day^d^		134,672 (29.8)	24,830 (26.6)	35,077 (36.2)	29,092 (32.0)	22,193 (24.5)	23,480 (29.4)
>6 to 12 g/day^e^		118,869 (26.3)	20,582 (22.1)	22,684 (23.4)	24,094 (26.5)	26,268 (29.0)	25,241 (31.6)
>12 to 24 g/day		70,605 (15.6)	9,983 (10.7)	11,582 (11.9)	15,617 (17.2)	18,802 (20.8)	14,621 (18.3)
>24 g/day		69,679 (15.4)	10,722 (11.5)	11,572 (11.9)	15,897 (17.5)	19,384 (21.4)	12,104 (15.2)
**Age at menarche, years** ^**f**^	308,875	13 ± 2	13 ± 2	13 ± 2	13 ± 2	13 ± 2	13 ± 2
**Age at menarche, categorical**	319,608						
≤12		112,773 (35.3)	24,712 (34)	22,956 (32.8)	24,193 (37.3)	21,682 (35.3)	19,230 (38)
13 to 14		147,378 (46.1)	35,312 (48.6)	30,411 (43.5)	29,788 (45.9)	28,802 (46.8)	23,065 (45.6)
≥15		48,724 (15.2)	11,819 (16.3)	10,540 (15.1)	9,468 (14.6)	9,959 (16.2)	6,938 (13.7)
Missing		10,733 (3.4)	848 (1.2)	5,987 (8.6)	1,492 (2.3)	1,045 (1.7)	1,361 (2.7)
**Age at first full-term pregnancy, years** ^**f**^	257,794	25 ± 4	25 ± 4	25 ± 4	25 ± 4	25 ± 4	25 ± 5
**Age at first full-term pregnancy, categorical** ^**f**^	305,652						
Nulliparous		46,945 (15.4)	7,464 (10.7)	7,575 (12)	13,923 (22.2)	9,212 (15.3)	8,771 (17.8)
≤21		57,190 (18.7)	15,898 (22.7)	11,969 (18.9)	9,438 (15)	11,405 (18.9)	8,480 (17.2)
22 to 30		174,342 (57)	41,164 (58.8)	38,160 (60.3)	33,742 (53.8)	34,278 (56.9)	26,998 (54.7)
>30		26,262 (8.6)	5,441 (7.8)	5,300 (8.4)	5,477 (8.7)	5,102 (8.5)	4,942 (10)
Missing		913 (0.3)	98 (0.1)	268 (0.4)	194 (0.3)	231 (0.4)	122 (0.2)
**Menopausal status** ^**f**^	319,608						
Premenopause		111,058 (34.7)	22,961 (31.6)	21,198 (30.3)	25,462 (39.2)	19,838 (32.3)	21,599 (42.7)
Perimenopause		63,049 (19.7)	18,185 (25)	15,546 (22.2)	11,050 (17)	10,994 (17.9)	7,274 (14.4)
Postmenopause		136,658 (42.8)	29,836 (41)	31,310 (44.8)	26,647 (41)	28,696 (46.7)	20,169 (39·9)
Surgical postmenopause		8,843 (2.8)	1,709 (2.4)	1,840 (2.6)	1,782 (2.7)	1,960 (3.2)	1,552 (3.1)
**Ever use of oral contraception, yes** ^**f**^	311,179	190,107 (61.1)	38,810 (53.6)	34,944 (54.4)	41,057 (64.3)	40,102 (65.8)	35,194 (70.9)
**Ever use of hormonal treatment for menopause (yes)** ^**f**^	297,860	80,471 (27)	17,781 (26.1)	15,272 (24.1)	14,901 (23.6)	18,733 (32.1)	13,784 (30.8)
**Deaths**	451,390	46,627 (10.3)	10,313 (11.1)	10,712 (11)	8,068 (8.9)	8,842 (9.8)	8,692 (10.9)
Age at death, years	46,627	71 (10)	71 (10)	70 (9)	70 (10)	71 (10)	74 (11)

^a^Values are median (*P*_10_–*P*_90_) for all dietary variables.

^b^Missing BMI for 3,710 (0.8%) and missing measured or self-reported height for 1,856 (0·4%) and weight for 3,361 (0·7%). When missing, height and weight were imputed with center-, age-, and gender-specific average values.

^c^Among first degree relatives.

^d^Among women, this category is >0 to 3 g/day.

^e^Among women, this category is >3 to 12 g/day.

^f^Among women only.

BMI, body mass index; DSR, dietary species richness; EPIC, European Prospective Investigation into Cancer and Nutrition; Q, quintile.

We performed several sensitivity analyses to test the robustness of our all-cause mortality results. First, we stratified our multivariable-adjusted models to estimate associations by sex and country (owing to the varying detail of food and drink items captured by DQs) separately. Furthermore, we removed Mediterranean diet score, red and processed meat, fiber, and total energy intake from the models to examine their role as mediators, rather than confounders. To assess the potential for residual confounding, we carried out subgroup analyses according to major potential categorical effect modifiers: educational level, smoking status, marital status, and physical activity. Furthermore, we used a “complete cases” approach, excluding participants with missing/unknown data on covariates. Not all food and drink items received a specific FoodEx2 species code, but rather kept a generic NCLASS classification (e.g., “other root vegetables,” which counted as one species toward an individual’s overall DSR). Therefore, we reran our models dropping these generic food and drink items. Analyses were also conducted including DSR without the lowest 5% and 10% of species intakes for each EPIC food group (see methodological reasoning above). In addition, we repeated our prospective analyses for species richness within each main EPIC food group adjusted for overall DSR (minus itself) to investigate whether one or more food groups were responsible for the observed associations [52]. Moreover, from the 46,627 fatal events, we excluded deaths within the first 3 (*n* = 2,969) and 6 years (*n* = 7,928) of follow-up to allow sufficient delay between baseline dietary assessment and mortality, thereby limiting reverse causality of subclinical disease. Findings from sensitivity analyses, which are not different (i.e., stable direction, strength, and trend of association) from those using the entire EPIC cohort, are not shown. To assess potential residual confounding from unmeasured or uncontrolled confounders, E-values were used [53,54].

All statistical tests were 2 sided, and *P* < 0.05 was considered statistically significant. *P*-values were adjusted for multiple testing of hypothesis using the Benjamini–Hochberg method. SAS software, version 9.4 (SAS Institute) was used for the analyses.

## Results

### Baseline characteristics

Initial characteristics from 451,390 eligible participants (71% female; median age 51 years) according to Qs of DSR are shown in Table 1. After a median follow-up of 17 years (7,506,482 person-years), 44,892 deaths from nonexternal causes occurred, among which 19,284 from cancer, 11,353 from diseases of the circulatory system, 2,479 from diseases of the respiratory system, and 1,386 from diseases of the digestive system. From the 11,858 items included in the EPIC food list, 80% were assigned FoodEx2 species codes (248 unique values; 78% of total kcal/day; see S1 Table), whereas 16% received a generic NCLASS code (100 unique values, e.g., “other citrus fruits”), and 4% were classified as “not applicable” (e.g., added salt and water). In the whole cohort, participant’s [median (*P*_10_–*P*_90_)] DSR was 68 (40 to 83) species per year. *Bos taurus* (cow), *Triticum aestivum* (common wheat grain), *Sus scrofa* (domestic pig), and *Solanum tuberosum* (potato) contributed most to self-reported total dietary energy intake (i.e., approximately 45%) with [mean % (SD)] 19% (8), 16% (8), 4% (3), and 4% (3) kcal/day, respectively. When comparing the fifth Q of DSR (highest; largely represented by the predominately vegetarian, “health-conscious” EPIC-Oxford (30%) and “omnivorous” German (35%) cohorts) against the first (lowest) Q, our findings indicate large differences in median dietary vegetable richness (22 versus 10 species), fruit, nuts, and seed richness (11 versus 5 species) and condiment richness (7 versus 2 species). In France, increased DSR across Qs was observed due to a significant positive gradient in vegetables richness only (7 versus 24 species).

### Food biodiversity and all-cause mortality

**Fig 2:**
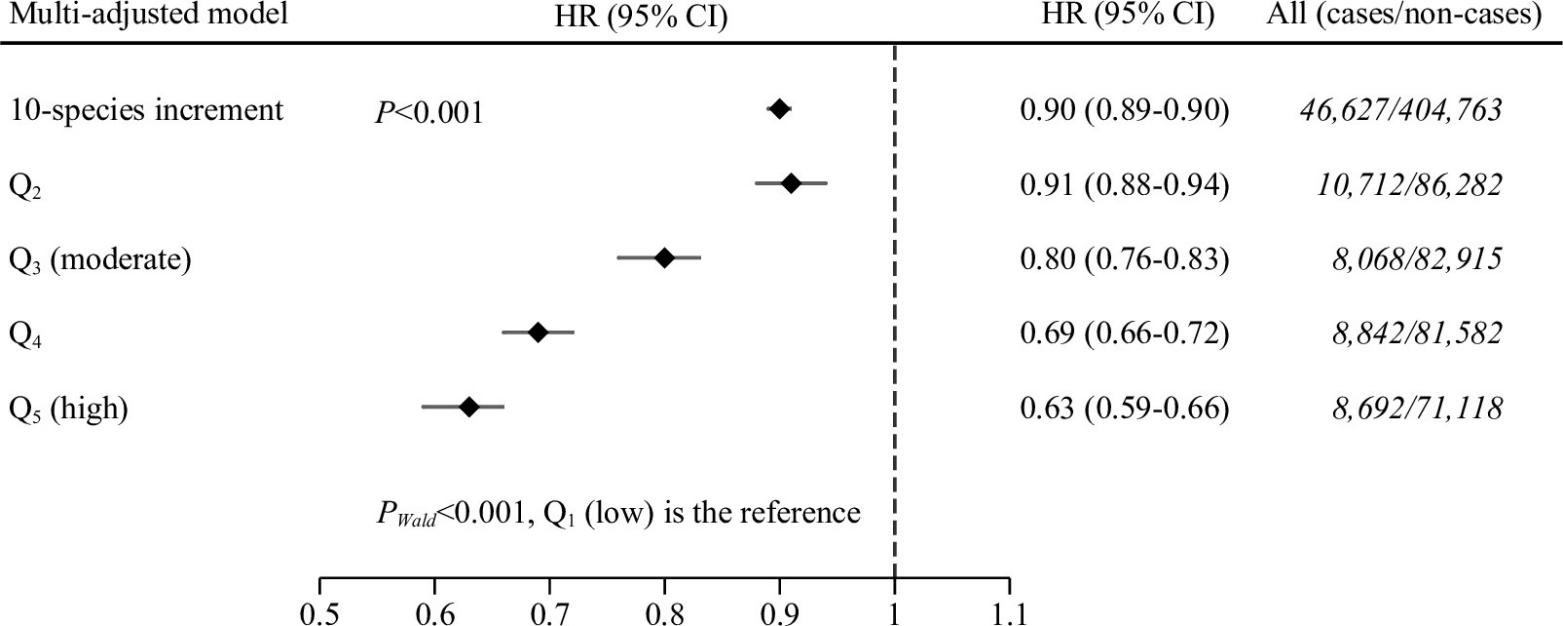
Inverse association between higher DSR per year and total mortality rate in the EPIC cohort, 1992 to 2014. Multiadjusted models were stratified for center, age at recruitment (1-year intervals, timescale), and sex and adjusted for baseline alcohol intake (g/day), physical activity (Cambridge index: active; moderately active; moderately inactive; inactive; missing), marital status (single, divorced, separated, or widowed; married or living together; unknown), smoking status and intensity of smoking (current, 1 to 15 cigarettes/day; current, 16 to 25 cigarettes/day; current, 26+ cigarettes/day; current, pipe/cigar/occasional; current/former, missing; former, quit 11 to 20 years; former, quit 20+ years; former, quit ≤10 years; never; unknown), educational level [longer education (including university degree, technical or professional school); secondary school; primary school completed; not specified], baseline energy intake (kcal/day), baseline fiber intake (g/day), baseline red and processed meat consumption (g/day), and an 18-point Mediterranean diet score [49]. *P*-values remained statistically significant after adjustment for multiple testing using the Benjamini–Hochberg method. CI, confidence interval; DSR, dietary species richness; EPIC, European Prospective Investigation into Cancer and Nutrition; HR, hazard ratio; Q, quintile.

Pooled multivariable analysis indicated that average DSR consumption was inversely associated with total mortality (*P*_*Wald*_ < 0.001 for trend), in that participants with low DSR (Q_1_; <48 species per year) had notably higher mortality rates than individuals with moderate (Q_3_; 64 to 72 species per year) or high DSR (Q_5_; ≥81 species per year) (see S2 Table). The corresponding pooled HRs (95% CIs) were 0·80 (0.76 to 0.83) for moderate DSR and 0.63 (0.59 to 0.66) for high DSR in comparison with low DSR (Fig 2). Absolute mortality rates among participants in the highest and lowest fifth of DSR were 65.4 and 69.3 cases/10,000 person-years, respectively (see S2 Table).

Our findings indicate slightly stronger relationships among males (see S3 Table). To illustrate, a 10-species increment in DSR was associated (95% CI) with a 14% to 17% and 6% to 8% reduction in all-cause mortality rates among males and females during approximately 20 years of follow-up, respectively. Overall, results for mortality rates from nonexternal causes were consistent across 8 countries (*P*-value and *P*_*Wald*_ both <0·05; see S4 Table). In the UK, we observed overall higher DSR and a subsequent smaller contrast between participants with lower and higher scores (Q_1_; <71, Q_5_; ≥82 species per year). The associated protective effect of DSR was not substantially changed when removing potential dietary mediators, among major subgroups (although the lowest HRs were reported among current smokers and participants with secondary education) or complete cases, when dropping generic food and drink codes, or exclusion of the lowest 5% (Q_1_; <47, Q_5_; ≥80 species per year) and 10% species intake (Q_1_; <46, Q_5_; ≥78 species per year) from each EPIC food (sub)group. Furthermore, our observed associations were not explained by species richness within one single food group, suggesting a positive cumulative effect of overall DSR (see S5 Table). Limited graphical, but statistically significant (*P*_*LR*_<0.001), evidence of nonlinearity was observed for all-cause mortality (S3 Fig).

### Food biodiversity and cause-specific mortality

In multivariable analyses, a 10-species increment in DSR was inversely associated with the rate of death [HR (95% CI)] due to digestive disease [0.80 (0.76 to 0.86)], respiratory disease [0.84 (0.80 to 0.88)], heart disease [0.88 (0.86 to 0.90)], and cancer [0.93 (0.92 to 0.95); all *P*_*Wald*_ < 0.001 for trend; Table 2].

**Table 2:** Associations between food biodiversity and cause-specific mortality rates from multivariable Cox proportional hazards regression models, EPIC cohort, 1992 to 2014.

			Qs of DSR					
	Per 10-species increment	*P*-value^c^	Q_1_	Q_2_	Q_3_	Q_4_	Q_5_	*P*-trend^c^
**DSR, species per year**			<48	[48 to 64]	[64 to 72]	[72 to 81]	≥81	
**Cancer**								
All (cases/person-years)	*19*,*284/7*,*506*,*482*		*4*,*335/1*,*577*,*991*	*4*,*385/1*,*662*,*237*	*3*,*621/1*,*532*,*349*	*3*,*860/1*,*479*,*202*	*3*,*083/1*,*254*,*703*	
Sex-adjusted model—HR (95% CI)^a^	0.89 (0.88 to 0.91)	<0.001	1.00 (ref)	0.87 (0.82 to 0.91)	0.77 (0.72 to 0.82)	0.67 (0.62 to 0.71)	0.64 (0.59 to 0.69)	<0.001
Multiadjusted model—HR (95% CI)^b^	0.93 (0.92 to 0.95)	<0.001	1.00 (ref)	0.92 (0.87 to 0.97)	0.87 (0.82 to 0.93)	0.78 (0.73 to 0.83)	0.75 (0.69 to 0.82)	<0.001
**CVD**								
All (cases/person-years)	*6*,*403/7*,*506*,*482*		*1*,*477/1*,*577*,*991*	*1*,*526/1*,*662*,*237*	*1*,*077/1*,*532*,*349*	*1*,*148/1*,*532*,*349*	*1*,*175/1*,*254*,*703*	
Sex-adjusted model—HR (95% CI)^a^	0.82 (0.80 to 0.84)	<0.001	1.00 (ref)	0.84 (0.77 to 0.92)	0.63 (0.56 to 0.70)	0.51 (0.45 to 0.58)	0.44 (0.38 to 0.50)	<0.001
Multiadjusted model—HR (95% CI)^b^	0.88 (0.85 to 0.91)	<0.001	1.00 (ref)	0.94 (0.86 to 1.03)	0.78 (0.69 to 0.87)	0.65 (0.57 to 0.74)	0.56 (0.49 to 0.65)	<0.001
**CHD**								
All (cases/person-years)	*4*,*950/7*,*506*,*482*		*1*,*195/1*,*577*,*991*	*1*,*081/1*,*662*,*237*	*679/1*,*532*,*349*	*873/1*,*532*,*349*	*1*,*122/1*,*254*,*703*	
Sex-adjusted model—HR (95% CI)^a^	0.77 (0.74 to 0.79)	<0.001	1.00 (ref)	0.72 (0.65 to 0.80)	0.52 (0.45 to 0.60)	0.46 (0.39 to 0.53)	0.35 (0.30 to 0.42)	<0.001
Multiadjusted model—HR (95% CI)^b^	0.87 (0.84 to 0.90)	<0.001	1.00 (ref)	0.89 (0.80 to 0.99)	0.75 (0.64 to 0.86)	0.67 (0.58 to 0.78)	0.55 (0.46 to 0.65)	<0.001
**Respiratory disease**								
All (cases/person-years)	*2*,*479/7*,*506*,*482*		*500/1*,*577*,*991*	*560/1*,*662*,*237*	*459/1*,*532*,*349*	*481/1*,*479*,*202*	*479/1*,*254*,*703*	
Sex-adjusted model—HR (95% CI)^a^	0.73 (0.70 to 0.76)	<0.001	1.00 (ref)	0.74 (0.64 to 0.86)	0.50 (0.41 to 0.60)	0.35 (0.29 to 0.42)	0.27 (0.21 to 0.34)	<0.001
Multiadjusted model—HR (95% CI)^b^	0.84 (0.80 to 0.88)	<0.001	1.00 (ref)	0.93 (0.80 to 1.09)	0.75 (0.62 to 0.91)	0.56 (0.46 to 0.69)	0.44 (0.34 to 0.55)	<0.001
**Digestive disease**								
All (cases/person-years)	*1*,*386/7*,*506*,*482*		*278/1*,*577*,*991*	*331/1*,*662*,*237*	*227/1*,*532*,*349*	*269/1*,*479*,*202*	*281/1*,*254*,*703*	
Sex-adjusted model—HR (95% CI)^a^	0.73 (0.69 to 0.77)	<0.001	1.00 (ref)	0.82 (0.67 to 1.01)	0.45 (0.35 to 0.58)	0.36 (0.28 to 0.47)	0.32 (0.23 to 0.42)	<0.001
Multiadjusted model—HR (95% CI)^b^	0.80 (0.76 to 0.86)	<0.001	1.00 (ref)	0.98 (0.80 to 1.21)	0.64 (0.49 to 0.83)	0.53 (0.41 to 0.69)	0.46 (0.34 to 0.63)	<0.001

^a^Sex-adjusted models were stratified for center, age at recruitment (1-year intervals, timescale), and sex.

^b^Multiadjusted models were stratified for center, age at recruitment (1-year intervals, timescale), and sex and adjusted for baseline alcohol intake (g/day), physical activity (Cambridge index: active; moderately active; moderately inactive; inactive; missing), marital status (single, divorced, separated, or widowed; married or living together; unknown), smoking status and intensity of smoking (current, 1 to 15 cigarettes/day; current, 16 to 25 cigarettes/day; current, 26+ cigarettes/day; current, pipe/cigar/occasional; current/former, missing; former, quit 11 to 20 years; former, quit 20+ years; former, quit ≤10 years; never; unknown), educational level [longer education (including university degree, technical or professional school); secondary school; primary school completed; not specified], baseline energy intake (kcal/day), baseline fiber intake (g/day), baseline red and processed meat consumption (g/day), and an 18-point Mediterranean diet score [49].

^c^*P*-values remained statistically significant after adjustment for multiple testing using the Benjamini–Hochberg method.

CHD, coronary heart disease; CI, confidence interval; CVD, cardiovascular disease; DSR, dietary species richness; EPIC, European Prospective Investigation into Cancer and Nutrition; HR, hazard ratio.

The large E-values for total and cause-specific mortality suggest that residual confounding is likely to be low, conditional on the measured covariates in our models (see S6 Table).

## Discussion

To our knowledge, this study is the first effort to investigate the relationships between food biodiversity and total and cause-specific mortality in a large epidemiological study. In the EPIC cohort, higher DSR was associated with reduced rates of total mortality and deaths due to cancer, heart disease, respiratory disease, and digestive disease, after accounting for sociodemographic, lifestyle, and other known dietary risk factors, which included relative Mediterranean diet score, red and processed meat, fiber, and total energy intake.

The mechanisms driving the observed relationships between DSR and human health may be largely due to 4 processes. The first is coined as the “sampling effect” and postulates that as one increases DSR, the greater the probability—simply by chance—of including (a diversity of) highly nutritious or health protective foods. In this regard, DSR might characterize both the substantial inter- and intra*-*food group variations, often not captured by other diet quality and diversity indicators [44], in the content and density of essential nutrients [26], bioactive nonnutrients, and anti-nutrients [55,56]. The second mechanism is known as the “complementary effect,” in which (chemical) interactions between species result in a function greater than expected by chance, i.e., each food or drink species might make an important contribution to diets, but none of these foods alone provide total “healthfulness” [46]. The third potential mechanism for the protective effect of higher food biodiversity encompasses “minimizing trade-offs,” which might occur from consuming too much of one single species (e.g., potential toxicity from overconsumption of certain fish species [57], cruciferous vegetables [58], Brazil nuts [59], and cassava [60]). Lastly, diet-induced variations in human microbial communities may contribute to metabolic health. To illustrate, the differences between the United States and Malawian or Amerindian gut microbiomes have been related to the differences in their diets, with a typical US diet being rich in (animal) protein, whereas diets in Malawi and Amerindian populations are dominated by corn and cassava [61].

In addition, to our knowledge, this was the first study to characterize usual DSR over an approximately 1-year time frame in a large multicountry cohort. There have been marked changes in the biodiversity landscape [62,63] and global food and agriculture supply/system in recent times [12]. To illustrate, retail level food availability data, rather than actual food intake assessments, estimated that in excess of half the global food energy (kcal/capita) is supplied by 4 staple crops: *Oryza* spp. (rice), *S*. *tuberosum* (potatoes), *Triticum* spp. (wheat), and *Zea mays* (maize) [64]. Our individual level self-reported dietary intake data from 9 diverse European populations, which includes consumer level waste and intra-household food distribution, suggest that animal species alone contributed over a quarter of total dietary energy, whereas the aforementioned staple crops also contributed a further 25% between 1992 and 2000 in the EPIC cohort. Our findings are alarming considering the growing realization that upstream agroforestry, aquatic, and other biosphere biodiversity loss, approximately 1 million species are now threatened with extinction [5], might have caused a further bottleneck of downstream consumer food choice [15] and thus have subsequent negative impacts on dietary (bio)diversity and food system sustainability [65].

The direct comparison between DSR and usual diet quality scores is neither straightforward nor warranted. Diet quality scores allocate points based on the consumption of specific complementary food items, food groups, or nutrients relevant for overall or specific chronic disease and mortality rates (e.g., Mediterranean diet score [66], WCRF/AICR adherence score [67], and Alternate Health Eating Index [68]), with the objective to add support to dietary recommendations and/or be a basis for food-based dietary guidelines. In contrast, DSR was not designed to find the best predictive score for total or cause-specific mortality rates; hence, our main analyses controlled for potential dietary confounders (i.e., established components of diet quality). Rather, we propose DSR as a simple crosscutting measure of 2 critical dimensions of sustainable development, i.e., human nutrition and biodiversity stewardship, which complements existing indicators for healthy and sustainable diets [26,69]. To maintain simplicity in DSR computation, we assigned an equal weight to each (rare or common) species consumed. Our approach thus fails to account for the relative abundance distribution of foods across a diet or species’ unique functional traits (see above). Similar to crude diet scores, DSR has inherent statistical limitations, including between and within food group species richness being considered as independent from one another (i.e., correlated structure of dietary components or substitution effects disregarded) and assumptions of linear additive effects [70]. No single intra-food group richness explained our main findings, which potentially clarifies the weaker associations in France, where only a strong positive gradient was observed across Qs for vegetable richness. Nevertheless, it remains unlikely that each species consumed made an equal contribution to the associated protective effect on mortality [27]. Thus, our objective was not to compare DSR to other existing dietary or food scores, as richness alone takes no account of the nutritional quality [71], degree of processing [72], and quantities of food and beverages consumed [73], but to specifically assess the relevance of the use of DSR in the framework of sustainable dietary recommendations and food-based dietary guidelines aiming to introduce “biodiversity/variety” into the European population [20]. Against the backdrop of anthropogenic species collapse [74] and rising dietary uniformity [12], our findings champion the relevance of food biodiversity, as a guiding principle of (inter)national food-based dietary guidelines, as explicitly included in, e.g., the Mediterranean Diet Pyramid [75], the New Nordic Diet [76], and Brazilian dietary recommendations [77].

Strengths of this study include its prospective design, large sample size, long (and high rates of) follow-up, and the inclusion of disease-free participants from different European countries with standardized data collection, especially for habitual diet, offering a broad and detailed perspective on a crosscutting measure of food biodiversity (approximately 250 unique species) in European diets. However, some limitations should be acknowledged. First, caution is needed regarding the extrapolation of these results to the entire European population or to other populations or ethnicities worldwide since this study included middle-aged volunteers from 9 European countries involved in a long-term cohort study investigating the association between nutrition and health, with overall more health-conscious behaviors compared to the general population. Therefore, individuals with lower DSR may have been underrepresented in this study, which may have weakened the observed inverse associations by inducing a smaller contrast between high and low DSR (or a potential food biodiversity threshold reached in the UK cohorts). Furthermore, in our models, we included all the participants with available dietary intake data, but with potential missing data on other covariates replaced with a “missing” class or imputation. Although this may have induced some bias, a “complete cases” model alone might have led to a selection bias toward more adherent participants in an already health-conscious population. Yet, our sensitivity analyses with complete cases provided similar results. In addition, this study used a single assessment of self-reported dietary intakes at baseline. Although diets may change over time, it is usually hypothesized that this estimation reflects general eating behavior throughout middle-aged adult life [78]. Traditional diet measurement instruments are built to capture the usual dietary intakes of an individual, but are still subject to imprecision and inaccuracy [79]. EPIC DQs consider self-reported usual food and drink intakes over longer periods of time, not the absolute number of species consumed per day or season specific dietary patterns. Hence, food items potentially consumed “less than once per month” or excluded during DQ development could not be counted toward DSR, which is hypothetically a source of underestimation. In addition, insufficient taxonomic detail was available to subdivide food and drink species into subspecies [e.g., *Triticum aestivum* subsp. *spelta* (spelt wheat)] or their source (e.g., locally produced or imported). Furthermore, the number of items that DQs cover depends on the country/center, which required in-depth standardization procedures to guarantee the comparability between countries. For all countries, recipes were decomposed into their ingredients using standard recipes. Therefore, herbs and spices and other ingredients potentially used in trivial amounts might have inflated the true value of an individual’s DSR. To best address this methodological limitation, we calculated 3 different scenarios of DSR consumption, namely overall DSR, including all food and drinks consumed in our EPIC food list (thus, also ingredients derived from standard recipes) and DSR, excluding the lowest 5% and 10% species intake from each EPIC food (sub)group. These sensitivity analyses confirmed the main analyses using overall DSR. Finally, this study was based on an observational cohort. Thus, even though EPIC included a large range of covariates, residual confounding in our models cannot be entirely ruled out (e.g., underlying inflammatory or metabolic disorders) [80] or unmeasured mediating pathways examined (e.g., role of gut microbiome). However, large E-values support the robustness of our observed DSR and mortality associations, providing support for the relationships having a causal basis.

In conclusion, the results from our analysis of a prospective study performed on a large Pan-European cohort with diverse profiles and dietary habits suggest that higher DSR is associated with lower rates of total and cause-specific mortality, independent from other known components of diet quality. Overall, this adds support to the relevance of public health and conservation measures advocating “dietary (species) biodiversity” aiming to influence the healthfulness at national and potentially supranational level. Future comparative and environmental impact (e.g., greenhouse gas emissions, land use, and water use) [39,81,82] studies may be carried out if other simple species diversity indicators with similar characteristics and a corresponding score derived at the individual level (e.g., capturing “optimal” species richness per food group) are to be proposed. In particular, this would complement strategies, such as food-based dietary guidelines [25], setting the basis for a diversified, environmentally sustainable diet mixing distinct types of food, both between and within food groups, and by highlighting food species for which a sensible consumption should be preferred for public and planetary health.

## Supporting information

S1 Fig Participants’ flowchart, EPIC cohort, 1992 to 2014. EPIC, European Prospective Investigation into Cancer and Nutrition.
(PDF)

S2 Fig Kaplan–Meier curve of overall survival probability by Q of DSR, EPIC cohort, 1992 to 2014. DSR, dietary species richness; EPIC, European Prospective Investigation into Cancer and Nutrition; Q, quintile.
(PDF)

S3 Fig Associations between food biodiversity and total mortality rate from multivariable Cox proportional hazards regression models using restricted cubic splines, EPIC cohort, 1992 to 2014. EPIC, European Prospective Investigation into Cancer and Nutrition.
(PDF)

S1 Table Food biodiversity codes assigned to the EPIC cohort’s food list. EPIC, European Prospective Investigation into Cancer and Nutrition.
(PDF)

S2 Table Associations between food biodiversity and total mortality rates from multivariable Cox proportional hazards regression models, EPIC cohort, 1992 to 2014. EPIC, European Prospective Investigation into Cancer and Nutrition.
(PDF)

S3 Table Associations between food biodiversity and total mortality rates from multivariable Cox proportional hazards regression models by sex, EPIC cohort, 1992 to 2014. EPIC, European Prospective Investigation into Cancer and Nutrition.
(PDF)

S4 Table Associations between food biodiversity and total mortality rates from multivariable Cox proportional hazards regression models by country, EPIC cohort, 1992 to 2014. EPIC, European Prospective Investigation into Cancer and Nutrition.
(PDF)

S5 Table Associations between food biodiversity, adjusted for species richness per food group, and total mortality rates from multivariable Cox proportional hazards regression model, EPIC cohort, 1992 to 2014. EPIC, European Prospective Investigation into Cancer and Nutrition.
(PDF)

S6 Table E-values for HR and 95% CI, associations between DSR and total and cause-specific mortality rates from multivariable Cox proportional hazards regression models, EPIC cohort, 1992 to 2014. CI, confidence interval; EPIC, European Prospective Investigation into Cancer and Nutrition; HR, hazard ratio.
(PDF)

S7 Table STROBE-nut: An Extension of the STROBE Statement for Nutritional Epidemiology. STROBE, STrengthening the Reporting of OBservational Studies in Epidemiology.
(PDF)

S1 Text Analysis plan extracted from the project proposal submitted to and approved (March, 2019) by the EPIC Steering Committee. EPIC, European Prospective Investigation into Cancer and Nutrition.
(PDF)

## Abbreviations

BMI: body mass index
CHD: coronary heart disease
CI: confidence interval
CVD: cardiovascular disease
DQ: dietary questionnaire
DSR: dietary species richness
EPIC: European Prospective Investigation into Cancer and Nutrition
FFQ: food frequency questionnaire
HR: hazard ratio
ICD-10: 10th revision of the International Statistical Classification of Diseases, Injuries, and Causes of Death
LMIC: low- and middle-income country
Q: quintile
SDG: Sustainable Development Goal
STROBE: STrengthening the Reporting of OBservational Studies in Epidemiology
WCRF: World Cancer Research Fund
