# Supplementary material for: Food biodiversity and total and cause-specific mortality in 9 European countries: An analysis of a prospective cohort study
Source: PLoS Med. 2021 Oct 18;18(10):e1003834. doi: 10.1371/journal.pmed.1003834 (PMC8559947; doi:10.1371/journal.pmed.1003834)

S1 Fig. Participants' flowchart, EPIC cohort, 1992–2014. EPIC, European Prospective Investigation into Cancer and Nutrition.

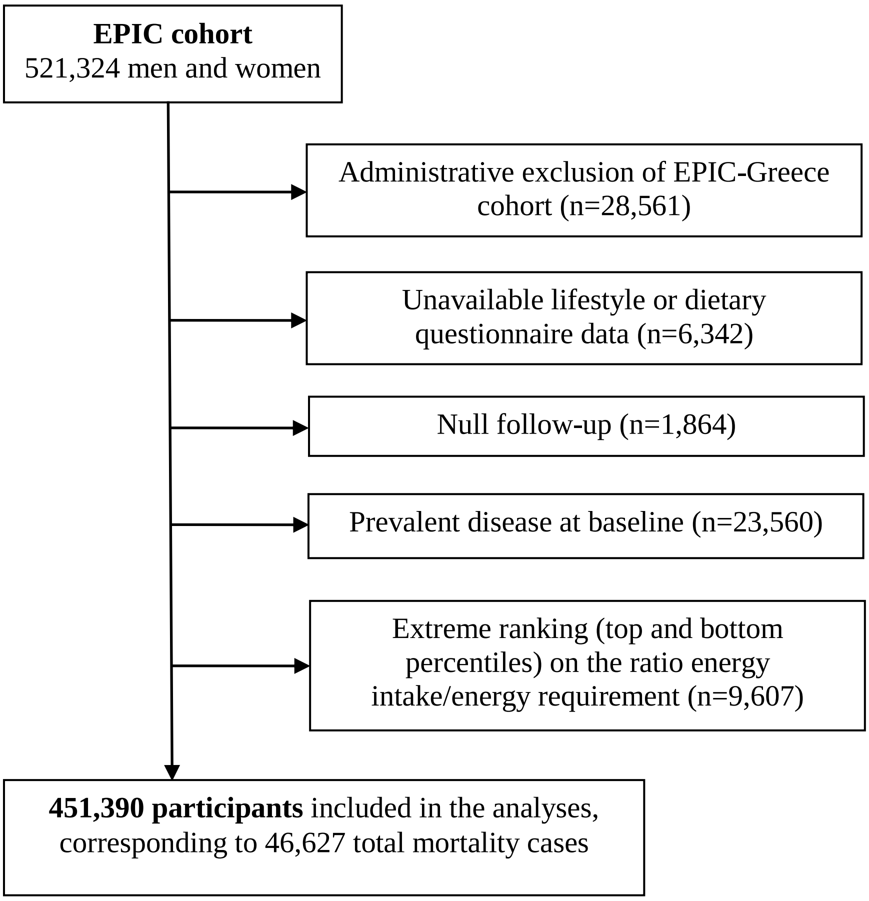

Supplement: S1 Fig — EPIC, European Prospective Investigation into Cancer and Nutrition. (PDF) [file pmed.1003834.s001.pdf]
