# Supplementary material for: Food biodiversity and total and cause-specific mortality in 9 European countries: An analysis of a prospective cohort study
Source: PLoS Med. 2021 Oct 18;18(10):e1003834. doi: 10.1371/journal.pmed.1003834 (PMC8559947; doi:10.1371/journal.pmed.1003834)

S2 Fig. Kaplan-Meier curve of overall survival probability by quintile of dietary species richness, EPIC cohort, 1992-2014. EPIC, European Prospective Investigation into Cancer and Nutrition

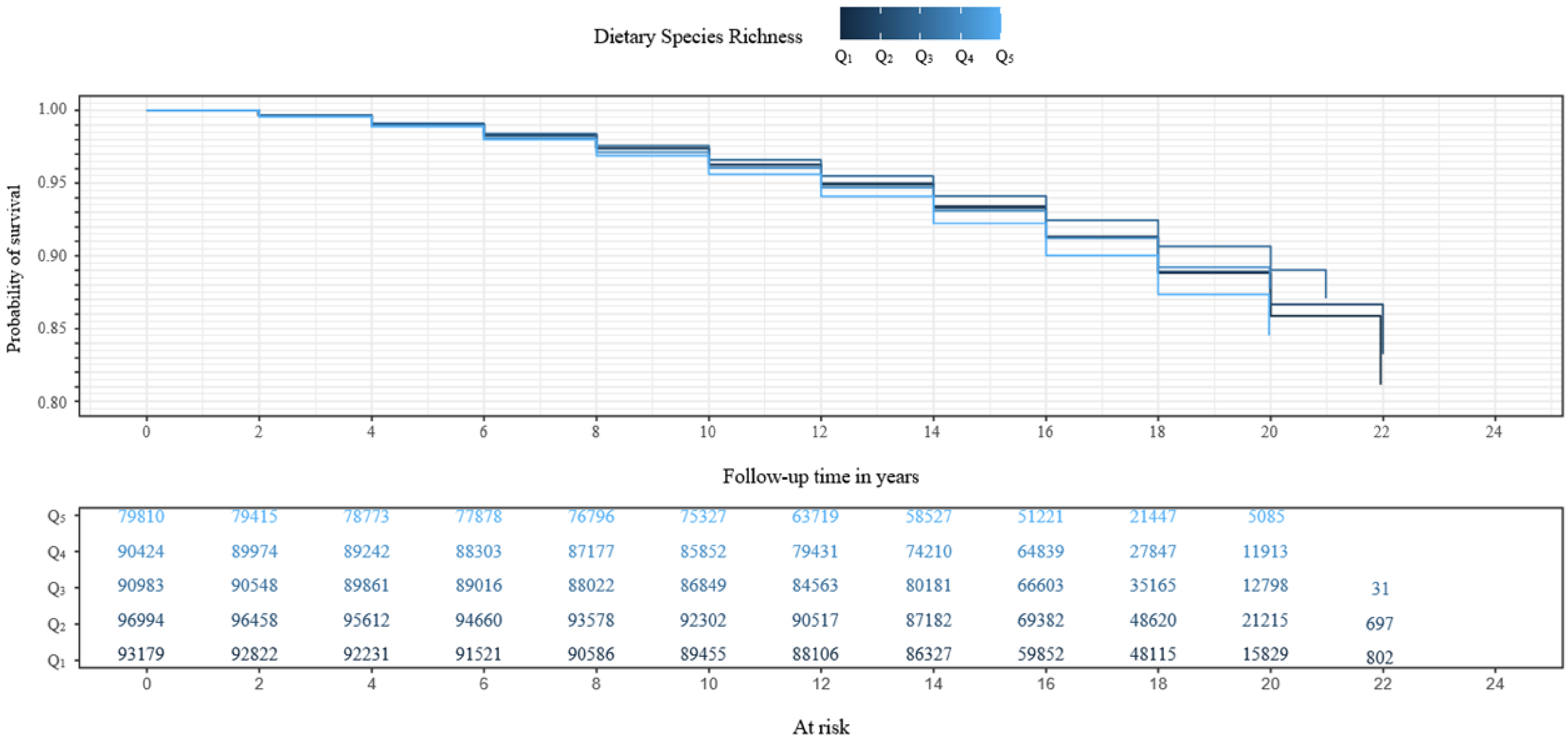

Supplement: S2 Fig — DSR, dietary species richness; EPIC, European Prospective Investigation into Cancer and Nutrition; Q, quintile. (PDF) [file pmed.1003834.s002.pdf]
