# Supplementary material for: Food biodiversity and total and cause-specific mortality in 9 European countries: An analysis of a prospective cohort study
Source: PLoS Med. 2021 Oct 18;18(10):e1003834. doi: 10.1371/journal.pmed.1003834 (PMC8559947; doi:10.1371/journal.pmed.1003834)
Supplement: S3 Fig — EPIC, European Prospective Investigation into Cancer and Nutrition. (PDF) [file pmed.1003834.s003.pdf]

S3 Fig. Associations between food biodiversity and total mortality rate from multivariable Cox proportional hazards regression models using restricted cubic splines, EPIC cohort, 1992-2014. EPIC, European Prospective Investigation into Cancer and Nutrition.

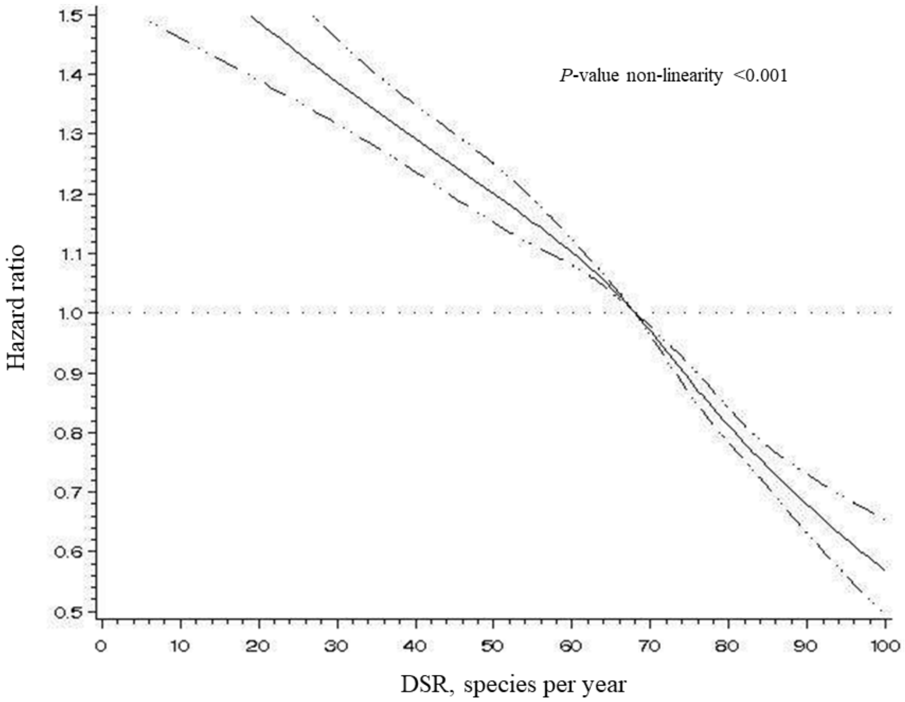

The black solid line represents the hazard ratio for all-cause mortality, modelled using restricted cubic splines with five knots set at 36, 55, 68, 77, and 88 species per year, respectively. The reference was set as the median (68 species per year). The black dashed lines represent the 95% confidence interval. Multi-adjusted model was stratified for centre, age at recruitment (1-y intervals, time-scale), and sex and adjusted for baseline alcohol intake (g/day), physical activity (Cambridge index: active; moderately active; moderately inactive; inactive; missing), marital status (single, divorced, separated, or widowed; married or living together; unknown), smoking status and intensity of smoking (current, 1-15 cigarettes/day; current, 16-25 cigarettes/day; current, 26+ cigarettes/day; current, pipe/cigar/occasional; current/former, missing; former, quit 11-20y; former, quit 20+y; former, quit ≤ 10y; never; unknown), educational level [longer education (incl. university degree, technical or professional school); secondary school; primary school completed; not specified], baseline energy intake (kcal/day), baseline fibre intake (g/day), baseline red and processed meat consumption (g/day), and an 18-point Mediterranean diet score [49].

Abbreviations: DSR, dietary species richness.
