# Supplementary material for: Food biodiversity and total and cause-specific mortality in 9 European countries: An analysis of a prospective cohort study
Source: PLoS Med. 2021 Oct 18;18(10):e1003834. doi: 10.1371/journal.pmed.1003834 (PMC8559947; doi:10.1371/journal.pmed.1003834)
Supplement: S1 Table — EPIC, European Prospective Investigation into Cancer and Nutrition. (PDF) [file pmed.1003834.s004.pdf]

S1 Table. Food biodiversity codes assigned to the EPIC cohort's food list. EPIC, European Prospective Investigation into Cancer and Nutrition.

FoodEx2 term code	Generic name	Species
A000G	Oat	*Avena sativa*
A000N	Buckwheat	*Fagopyrum esculentum*
A000P	Barley	*Hordeum vulgare*
A000T	Maize	*Zea mays*
A000Y	Finger millet	*Eleusine coracana*
A001B	Pearl millet	*Cenchrus americanus*
A001D	Asian rice	*Oryza sativa*
A001K	Rye	*Secale cereale*
A001N	Common wheat, spelt	*Triticum aestivum*
A001P	Durum wheat	*Triticum durum*
A00FX	Head cabbages	*Brassica oleracea*
A00GG	Chinese cabbages, turnip	*Brassica rapa*
A00GZ	Garlic	*Allium sativum*
A00HC	Onions	*Allium cepa*
A00JA	Sweet pepper	*Capsicum annuum*
A00JB	Chili peppers	*Capsicum frutescens*
A00JD	Aubergine	*Solanum melongena*
A00JF	Okra	*Abelmoschus esculentus*
A00JM	Cucumber, gherkin	*Cucumis sativus*
A00KA	Burr gherkin	*Cucumis anguria*
A00JR	Courgette, pumpkin, squash	*Cucurbita pepo*
A00KF	Melons	*Cucumis melo*
A00KJ	Watermelon	*Citrullus lanatus*
A00KX	Head lettuces	*Lactuca sativa*
A00LF	Curly endives	*Cichorium endivia*
A00LG	Radicchio, Belgian endives	*Cichorium intybus*
A00LQ	Red mustard leaves	*Brassica juncea*
A00MJ	Spinach	*Spinacia oleracea*
A00ND	Watercress	*Nasturtium officinale*
A00NQ	Salsify leaves	*Tragopogon porrifolius*
A00PA	Common nettle	*Urtica dioica*
A00PH	Broad beans	*Vicia faba*
A00PR	Mung beans	*Vigna radiata*
A00PV	Black eyed peas	*Vigna unguiculata*
A00PY	Peas	*Pisum sativum*
A00QG	Beets, chards	*Beta vulgaris*
A00QH	Carrots	*Daucus carota*
A00QJ	Celeriac, celery	*Apium graveolens*
A00QK	Horseradish root	*Armoracia rusticana*
A00QQ	Jerusalem artichoke	*Helianthus tuberosus*
A00QR	Parsnip root	*Pastinaca sativa*
A00QV	Radish, daikon	*Raphanus sativus*
A00RC	Black salsify	*Scorzonera hispanica*
A00RD	Swede, rapeseed	*Brassica napus*
A00RS	Globe artichoke, cardoon	*Cynara cardunculus*
A00RT	Asparagus	*Asparagus officinalis*
A00RV	Bamboo	*Bambusa vulgaris*
A00SB	Leek	*Allium ampeloprasum*
A00SD	Rhubarb	*Rheum rhabarbarum*
A00SH	Alfalfa	*Medicago sativa*
A00TQ	Button mushrooms	*Agaricus bisporus*
A00VD	Sea lettuce	*Ulva lactuca*
A00VH	Laver	*Porphyra yezoensis*
A00VL	Hijiki	*Sargassum fusiforme*
A00VV	Basil	*Ocimum basilicum*
A00XD	Chive	*Allium schoenoprasum*
A00XV	Oregano	*Origanum vulgare*
A00YE	Parsley	*Petroselinum crispum*
A00ZT	Potato	*Solanum tuberosum*

A00ZZ	Cassava	*Manihot esculenta*
A010C	Sweet potato	*Ipomoea batatas*
A012F	Soybean	*Glycine max*
A012T	Common beans	*Phaseolus vulgaris*
A013M	Chickpea	*Cicer arietinum*
A013Q	Lentil	*Lens culinaris*
A013S	White lupine	*Lupinus albus*
A013V	Chickling vetches	*Lathyrus sativus*
A014D	Almond	*Amygdalus communis*
A014H	Cashew nut	*Anacardium occidentale*
A014J	Chestnuts	*Castanea* spp.
A014K	Coconut	*Cocos nucifera*
A014L	Hazelnut	*Corylus avellana*
A014N	Pecan	*Carya illinoinensis*
A014Q	Pistachio	*Pistacia vera*
A014R	Walnut	*Juglans regia*
A015G	Linseed	*Linum usitatissimum*
A015H	Peanut	*Arachis hypogaea*
A015K	Sesame	*Sesamum indicum*
A015L	Sunflower	*Helianthus annuus*
A015S	Mustard seeds	*Sinapis* spp.
A015Y	Safflower seeds	*Carthamus tinctorius*
A016P	Oil palms	*Elaeis guineensis*
A017J	Rosemary	*Salvia rosmarinus*
A017Q	Thyme	*Thymus vulagris*
A017Y	Anise seed	*Pimpinella anisum*
A018F	Dill	*Anethum graveolens*
A018G	Fennel	*Foeniculum vulgare*
A018Z	Peppercorn	*Piper nigrum*
A019V	Cinnamon bark	*Cinnamomum verum*
A01AM	Caper	*Capparis spinosa*
A01AR	Saffron	*Crocus sativus*
A01BP	Olive	*Olea europaea*
A01BV	Pine nuts	*Pinus* spp.
A01BY	Lemon	*Citrus limon*
A01CA	Lime	*Citrus aurantifolia*
A01CD	Mandarins, clementine	*Citrus reticulata*
A01CY	Grapefruits	*Citrus paradisi*
A01DJ	Apples	*Malus domestica*
A01DL	Loquats	*Eriobotrya japonica*
A01DP	Pear	*Pyrus communis*
A01DX	Grapes	*Vitis vinifera*
A01EA	Strawberry	*Fragaria x ananassa Duchene ex Rozier*
A01EE	Blackberry	*Rubus fruticosus*
A01EM	Cloudberry	*Rubus chamaemorus*
A01EP	Raspberries	*Rubus idaeus*
A01EY	Blueberries	*Vaccinium corymbosum*
A01FH	Cranberries	*Vaccinium macrocarpon*
A01FK	Cowberries, lingonberries	*Vaccinium vitis*
A01FM	Redcurrant	*Ribes rubrum*
A01FN	Blackcurrant	*Ribes nigrum*
A01GF	Apricots	*Armeniaca vulgaris*
A01GH	Sour cherry	*Prunus cerasus*
A01GK	Sweet cherry	*Prunus avium*
A01GM	Common peaches	*Prunus persica*
A01GN	Nectarines	*Persica vulgaris*
A01GQ	Plum	*Prunus domestica*
A01HF	Date	*Phoenix dactylifera*
A01HG	Fig	*Ficus carica*
A01HQ	Kaki	*Diospyros kaki*
A01JT	Kiwi	*Actinidia Lindl.* spp.
A01KC	Passionfruit	*Passiflora edulis*
A01KD	Prickly pear	*Opuntia ficus-indica*

A01LB	Avocado	*Persea americana*
A01LC	Common banana, plantain	*Musa acuminata*
A01LF	Mango	*Mangifera indica*
A01LG	Papayas	*Carica papaya*
A01LH	Pomegranate	*Punica granatum*
A01LJ	Cherimoya	*Annona cherimola*
A01LP	Pineapple	*Ananas comosus*
A01QV	Cow	*Bos taurus*
A01QZ	Buffalo	*Bubalus* spp.
A01RG	Pig, boar	*Sus scrofa*
A01RH	Sheep	*Ovis aries*
A01RL	Goat	*Capra aegagrus*
A01RM	Horse	*Equus ferus*
A01RQ	Rabbit	*Oryctolagus cuniculus*
A01SA	Red deer	*Cervus elaphus*
A01SE	European moose	*Alces alces*
A01SF	Reindeer	*Rangifer tarandus*
A01SG	Roe deer	*Capreolus capreolus*
A01SJ	Hare	*Lepus europaeus*
A01SP	Chicken	*Gallus gallus*
A01SQ	Turkey	*Meleagris gallopavo*
A01SR	Duck	*Anas platyrhynchos*
A01SS	Goose	*Anser anser*
A01SZ	Pheasant	*Phasianus colchicus*
A01TA	Pigeon	*Columba livia*
A01TB	Quail	*Coturnix coturnix*
A01TK	Ptarmigan	*Lagopus muta*
A027B	European freshwater bream	*Abramis brama*
A027Q	European perch	*Perca fluviatilis*
A027X	Northern pike	*Esox lucius*
A027Y	Pike-perch	*Stizostedium lucioperca*
A028K	European eel	*Anguilla anguilla*
A028P	Atlantic salmon	*Salmo salar*
A028X	Pink salmon	*Oncorhynchus gorbuscha*
A029B	Smelt	*Osmerus eperlanus*
A029E	Sturgeon	*Huso huso*
A029F	Trout	*Oncorhynchus clarkii*
A029G	Arctic char	*Salvelinus alpinus*
A029N	Rainbow trout	*Salmo gairdneri*
A029Q	Whitefish, *Coregonus*	*Coregonus* spp.
A029T	Sea bass	*Morone labrax*
A029V	Sea bream	*Pagellus centrodontus*
A029Z	Wolffish	*Anarhichas lupus*
A02AD	Mullets	*Mugil cephalus*
A02AL	European conger	*Conger conger*
A02AN	Scorpion fish	*Scorpaena dactyloptera*
A02AP	Atlantic pomfret	*Brama brama*
A02AS	Common dab	*Limanda limanda*
A02AT	Flounders	*Platichthys flesus*
A02AZ	Atlantic halibut	*Hippoglossus hippoglossus*
A02BE	European plaice	*Pleuronectes platessa*
A02BF	Sole	*Solea solea*
A02BG	Turbot	*Scophthalmus maximus*
A02BJ	Monkfish, anglerfish	*Lophius* spp.
A02BS	Garfish	*Belone belone*
A02BV	Cod	*Gadus callarias*
A02CB	Hakes	*Merluccius* spp.
A02CC	Ling	*Molva molva*
A02CF	Coalfish	*Pollachius virens*
A02CG	Whiting	*Gadus merlangus*
A02CJ	Norway pout	*Trisopterus esmarkii*
A02CN	Horse mackerel	*Trachurus capensis*
A02CV	Atlantic mackerel	*Scomber scombrus*
A02DB	European sardine	*Sardina pilchardus Walbaum*

A02DD	Anchovies	*Engraulis encrasicolus*
A02DF	Herrings	*Clupea harengus*
A02DH	European sprat	*Sprattus sprattus*
A02DN	Smooth hounds	*Mustelus* spp.
A02DP	Spiny dogfish	*Squalus acanthias*
A02DQ	Common skate	*Dipturus batis*
A02DS	Bonito	*Sarda chilensis*
A02DX	Tuna	*Thunnus alalunga*
A02EF	Skipjack tuna	*Katsuwonus pelamis*
A02FG	European freshwater crayfish	*Astacus astacus*
A02FK	Barnacle	*Megabalanus tintinnabulum*
A02FL	European spider crab	*Maja squinado*
A02FP	American lobster	*Homarus americanus*
A02FS	Norwegian lobster	*Nephrops norvegicus*
A02FV	Spiny lobster	*Palinurus elephas*
A02FY	Giant tiger prawn	*Penaeus monodon*
A02FZ	Northern prawn	*Pandalus borealis*
A02GB	Common shrimps	*Crangon crangon*
A02GQ	Purple urchin	*Paracentrotus lividus*
A02GS	Common periwinkle	*Littorina littorea*
A02HA	Smoot clam	*Callista chione*
A02HB	Razor clam	*Solen marginatus*
A02HE	Cockles	*Cardium edule*
A02HF	Common mussel	*Mytilus edulis*
A02HJ	European oyster	*Ostrea edulis*
A02JB	Cuttlefishes	*Sepia officinalis*
A02JE	Octopuses	*Octopus vulgaris*
A02JJ	Squids	*Loligo forbesi*
A02LL	Garden snail	*Helix aspersa*
A02LN	Roman snail	*Helix pomatia*
A03GK	Coffee bean	*Coffea liberica*
A03HF	Cocoa bean	*Theobroma cacao*
A048Z	Yeast cultures	*Saccharomyces* spp.
A04KK	Tea leaves	*Camellia sinensis*
A07Y0	Swordfish	*Xiphias gladius*
A0CFN	Gooseberry	*Ribes uva-crispa*
A0CFR	Rose hips	*Rosa* spp·
A0CGX	Ginger	*Zingiber officinale*
A0DER	Borage	*Borago officinalis*
A0DLT	Butternut squashes	*Cucurbita moschata*
A0DMX	Tomatoes	*Solanum lycopersicum*
A0DZB	Oranges	*Citrus sinensis*
A0F7F	Lumpfish	*Cyclopterus lumpus*
A0F9K	Marble goby	*Oxyeleotris marmorata*
A0FAL	Megrims	*Lepidorhombus whiffiagonis*
A0FAM	Pollock	*Pollachius pollachius*
A0FAN	Blue whiting	*Micromesistius poutassou*
A0FAP	Alaska pollock	*Theragra chalcogramma*
A0FAR	Gilthead seabream	*Sparus aurata*
A0FAS	Groupers	*Epinephelus* spp.
A0FBD	Sea catfish	*Bagre marinus*
A0FBH	Alfonsino	*Beryx* spp.
A0FBN	Amberjack	*Seriola* spp.
A0FBQ	Blue shark	*Prionace glauca*
A0FCP	Edible crab	*Cancer pagurus*
A170Z	Rockweed	*Ascophyllum nodosum*
