# Supplementary material for: Food biodiversity and total and cause-specific mortality in 9 European countries: An analysis of a prospective cohort study
Source: PLoS Med. 2021 Oct 18;18(10):e1003834. doi: 10.1371/journal.pmed.1003834 (PMC8559947; doi:10.1371/journal.pmed.1003834)
Supplement: S2 Table — EPIC, European Prospective Investigation into Cancer and Nutrition. (PDF) [file pmed.1003834.s005.pdf]

S2 Table. Associations between food biodiversity and total mortality rates from multivariable Cox proportional hazards regression models, EPIC cohort, 1992-2014. EPIC, European Prospective Investigation into Cancer and Nutrition.

			**Quintiles (Qs) of dietary species richness (DSR)**					
	**Per 10-species increment**	***P*-value**	**Q1**	**Q2**	**Q3**	**Q4**	**Q5**	***P*-trend**
**DSR, species per year**			<48	[48 – 64]	[64 – 72]	[72 – 81]	≥81	
**Total mortality**								
All (cases/person-years)	*46,627/7,506,482*		*10,313/1,577,991*	*10,712/1,662,237*	*8,068/1,532,349*	*8,842/1,479,202*	*8,692/1,254,703*	
Sex-adjusted model - HR (95% CI)[a]	0·84 (0·83-0·85)	<0·001	1·00 (ref)	0·83 (0·80-0·86)	0·67 (0·64-0·70)	0·56 (0·54-0·58)	0·50 (0·47-0·52)	<0·001
Multi-adjusted model - HR (95% CI)[b]	0·90 (0·89-0·90)	<0·001	1·00 (ref)	0·91 (0·88-0·94)	0·80 (0·76-0·83)	0·69 (0·66-0·72)	0·63 (0·59-0·66)	<0·001

[a]Sex-adjusted models were stratified for centre, age at recruitment (1-y intervals, time-scale), and sex.
[b]Multi-adjusted models were stratified for centre, age at recruitment (1-y intervals, time-scale), and sex and adjusted for baseline alcohol intake (g/day), physical activity (Cambridge index: active; moderately active; moderately inactive; inactive; missing), marital status (single, divorced, separated, or widowed; married or living together; unknown), smoking status and intensity of smoking (current, 1-15 cigarettes/day; current, 16-25 cigarettes/day; current, 26+ cigarettes/day; current, pipe/cigar/occasional; current/former, missing; former, quit 11-20y; former, quit 20+y; former, quit ≤ 10y; never; unknown), educational level [longer education (incl. university degree, technical or professional school); secondary school; primary school completed; not specified], baseline energy intake (kcal/day), baseline fibre intake (g/day), baseline red and processed meat consumption (g/day), and an 18-point Mediterranean diet score [49].
Abbreviations: CI, confidence interval; HR, hazard ratio.
