# Supplementary material for: Food biodiversity and total and cause-specific mortality in 9 European countries: An analysis of a prospective cohort study
Source: PLoS Med. 2021 Oct 18;18(10):e1003834. doi: 10.1371/journal.pmed.1003834 (PMC8559947; doi:10.1371/journal.pmed.1003834)
Supplement: S3 Table — EPIC, European Prospective Investigation into Cancer and Nutrition. (PDF) [file pmed.1003834.s006.pdf]

S3 Table. Associations between food biodiversity and total mortality rates from multivariable Cox proportional hazards regression models by sex, EPIC cohort, 1992-2014. EPIC, European Prospective Investigation into Cancer and Nutrition.

				**Quintiles (Qs) of dietary species richness (DSR)**					
	**N**	**Per 10-species increment**	***P*-value**	**Q_1**	**Q_2**	**Q_3**	**Q_4**	**Q_5**	***P*-trend**
**Female**	319,608								
DSR, species per year				<47	[47 – 62]	[62 – 71]	[71 – 80]	≥80	
All (cases/person-years)		*25,211/5,374,647*		*5,233/1,145,668*	*5,366/1,040,700*	*4,978/1,132,122*	*4,618/1,114,676*	*5,016/941,482*	
Multi-adjusted model - HR (95% CI)[a]		0·93 (0·92-0·94)	<0·001	1·00 (ref)	0·94 (0·90-0·98)	0·87 (0·82-0·91)	0·77 (0·73-0·82)	0·73 (0·68-0·78)	<0·001
**Male**	131,782								
DSR, species per year				<56	[56 – 67]	[67 – 74]	[74 – 82]	≥82	
All (cases/person-years)		*21,416/2,131,834*		*6,650/515,076*	*3,883/440,477*	*2,979/383,975*	*5,969/523,579*	*1,935/268,728*	
Multi-adjusted model - HR (95% CI)[a]		0·84 (0·83-0·86)	<0·001	1·00 (ref)	0·78 (0·74-0·83)	0·63 (0·59-0·67)	0·58 (0·54-0·62)	0·50 (0·46-0·54)	<0·001

[a]Multi-adjusted models were stratified for centre and age at recruitment (1-y intervals, time-scale) and adjusted for baseline alcohol intake (g/day), physical activity (Cambridge index: active; moderately active; moderately inactive; inactive; missing), marital status (single, divorced, separated, or widowed; married or living together; unknown), smoking status and intensity of smoking (current, 1-15 cigarettes/day; current, 16-25 cigarettes/day; current, 26+ cigarettes/day; current, pipe/cigar/occasional; current/former, missing; former, quit 11-20y; former, quit 20+y; former, quit ≤ 10y; never; unknown), educational level [longer education (incl. university degree, technical or professional school); secondary school; primary school completed; not specified], baseline energy intake (kcal/day), baseline fibre intake (g/day), baseline red and processed meat consumption (g/day), and an 18-point Mediterranean diet score [49].

Abbreviations: CI, confidence interval; HR, hazard ratio.
