# Supplementary material for: Food biodiversity and total and cause-specific mortality in 9 European countries: An analysis of a prospective cohort study
Source: PLoS Med. 2021 Oct 18;18(10):e1003834. doi: 10.1371/journal.pmed.1003834 (PMC8559947; doi:10.1371/journal.pmed.1003834)
Supplement: S4 Table — EPIC, European Prospective Investigation into Cancer and Nutrition. (PDF) [file pmed.1003834.s007.pdf]

S4 Table. Associations between food biodiversity and total mortality rates from multivariable Cox proportional hazards regression models by country, EPIC cohort, 1992-2014. EPIC, European Prospective Investigation into Cancer and Nutrition.

			Quintiles (Qs) of dietary species richness (DSR)					
	**Per 10-species increment**	***P*-value**	**Q_1**	**Q_2**	**Q_3**	**Q_4**	**Q_5**	***P*-trend**
**Denmark**								
DSR, species per year			<67	[67 – 72]	[72 – 75]	[75 – 78]	≥78	
All (cases/person-years)	*9,418/888,078*		*3,494/216,480*	*2,010/194,836*	*1,395/158,601*	*1,447/172,529*	*1,072/145,631*	
Multi-adjusted model - HR (95% CI)[a]	0·82 (0·80-0·84)	<0·001	1·00 (ref)	0·76 (0·72-0·81)	0·70 (0·65-0·74)	0·69 (0·65-0·74)	0·65 (0·61-0·70)	<0·001
**France**								
DSR, species per year			<42	[42 – 62]	[62 – 70]	[70 – 76]	≥76	
All (cases/person-years)	*4,455/1,303,948*		*1,049/265,399*	*1,107/270,743*	*867/284,128*	*728/244,715*	*704/238,963*	
Multi-adjusted model - HR (95% CI)[a]	0·98 (0·96-1·00)	0·03	1·00 (ref)	1·01 (0·93-1·11)	0·86 (0·78-0·94)	0·92 (0·83-1·01)	0·96 (0·86-1·06)	0·03
**Germany**								
DSR, species per year			<76	[76 – 81]	[81 – 84]	[84 – 88]	≥88	
All (cases/person-years)	*3,430/674,650*		*1,263/149,179*	*818/145,049*	*535/121,233*	*530/158,866*	*264/100,323*	
Multi-adjusted model - HR (95% CI)[a]	0·85 (0·82-0·89)	<0·001	1·00 (ref)	0·88 (0·80-0·96)	0·81 (0·73-0·90)	0·73 (0·66-0·82)	0·64 (0·56-0·74)	<0·001
**Italy**								
DSR, species per year			<61	[61 – 67]	[67 – 71]	[71 – 76]	≥76	
All (cases/person-years)	*2,423/689,933*		*663/142,376*	*585/151,359*	*389/126,995*	*451/146,004*	*335/123,199*	
Multi-adjusted model - HR (95% CI)[a]	0·89 (0·85-0·94)	<0·001	1·00 (ref)	0·95 (0·85-1·07)	0·78 (0·68-0·89)	0·86 (0·75-0·98)	0·78 (0·66-0·92)	<0·001
**the Netherlands**								
DSR, species per year			<60	[60 – 65]	[65 – 68]	[68 – 71]	≥71	
All (cases/person-years)	*3,471/610,475*		*1,343/140,988*	*918/144,492*	*525/113,244*	*432/114,368*	*253/97,383*	
Multi-adjusted model - HR (95% CI)[a]	0·86 (0·82-0·91)	<0·001	1·00 (ref)	0·93 (0·86-1·02)	0·87 (0·78-0·96)	0·82 (0·73-0·92)	0·69 (0·59-0·79)	<0·001
**Norway**								
DSR, species per year			<35	[35 – 40]	[40 – 44]	[44 – 49]	≥49	
All (cases/person-years)	*1,138 /471,145*		*349/112,099*	*209/80,461*	*230/97,296*	*203/91,479*	*147/89,810*	
Multi-adjusted model - HR (95% CI)[a]	0·77 (0·69-0·86)	<0·001	1·00 (ref)	0·92 (0·77-1·10)	0·89 (0·75-1·07)	0·88 (0·73-1·07)	0·66 (0·53-0·82)	<0·001
**Spain**								

DSR, species per year			<35	[35 – 40]	[40 – 44]	[44 – 49]	≥49	
All (cases/person-years)	*3,429/737,975*		*1,006/163,920*	*780/158,648*	*609/140,311*	*556/139,988*	*478/135,107*	
Multi-adjusted model - HR (95% CI)[a]	0·88 (0·83-0·92)	<0·001	1·00 (ref)	0·88 (0·80-0·97)	0·82 (0·73-0·91)	0·78 (0·70-0·88)	0·75 (0·66-0·85)	<0·001
**Sweden**								
DSR, species per year			<45	[45 – 54]	[54 – 60]	[60 – 63]	≥63	
All (cases/person-years)	*8,633/878,162*		*3,693/212,320*	*2,226/192,489,*	*1,170/169,380*	*844/162,753*	*700/141,220*	
Multi-adjusted model - HR (95% CI)[a]	0·89 (0·87-0·92)	<0·001	1·00 (ref)	0·91 (0·86-0·96)	0·83 (0·76-0·90)	0·80 (0·72-0·89)	0·75 (0·67-0·84)	<0·001
**the United Kingdom**								
DSR, species per year			<71	[71 – 76]	[76 – 81]	[81 – 82]	≥82	
All (cases/person-years)	*3,904/760,356*		*714/160,357*	*613/152,542*	*910/152,991*	*859/151,662*	*808/142,804*	
Multi-adjusted model - HR (95% CI)[a]	0·96 (0·90-1·02)	0·20	1·00 (ref)	1·03 (0·92-1·15)	0·97 (0·87-1·09)	0·91 (0·81-1·03)	0·92 (0·82-1·04)	0·03

[a]Multi-adjusted models were stratified for centre, age at recruitment (1-y intervals, time-scale), and sex and adjusted for baseline alcohol intake (g/day), physical activity (Cambridge index: active; moderately active; moderately inactive; inactive; missing), marital status (single, divorced, separated, or widowed; married or living together; unknown), smoking status and intensity of smoking (current, 1-15 cigarettes/day; current, 16-25 cigarettes/day; current, 26+ cigarettes/day; current, pipe/cigar/occasional; current/former, missing; former, quit 11-20y; former, quit 20+y; former, quit ≤ 10y; never; unknown), educational level [longer education (incl. university degree, technical or professional school); secondary school; primary school completed; not specified], baseline energy intake (kcal/day), baseline fibre intake (g/day), baseline red and processed meat consumption (g/day), and an 18-point Mediterranean diet score [49].

Abbreviations: CI, confidence interval; HR, hazard ratio.
