# Supplementary material for: Food biodiversity and total and cause-specific mortality in 9 European countries: An analysis of a prospective cohort study
Source: PLoS Med. 2021 Oct 18;18(10):e1003834. doi: 10.1371/journal.pmed.1003834 (PMC8559947; doi:10.1371/journal.pmed.1003834)
Supplement: S5 Table — EPIC, European Prospective Investigation into Cancer and Nutrition. (PDF) [file pmed.1003834.s008.pdf]

S5 Table. Associations between food biodiversity, adjusted for species richness per food group, and total mortality rates from multivariable Cox proportional hazards regression models, EPIC cohort, 1992-2014. EPIC, European Prospective Investigation into Cancer and Nutrition.

	Per 10-species increment	*P*-value	Δ HR
**DSR, minus tuber species**			
Multi-adjusted model - HR (95% CI)[a]	0·91 (0·90-0·92)	<0·001	1%
**DSR, minus vegetable species**			
Multi-adjusted model - HR (95% CI)[a]	0·88 (0·87-0·89)	<0·001	-2%
**DSR, minus legume species**			
Multi-adjusted model - HR (95% CI)[a]	0·91 (0·90-0·92)	<0·001	1%
**DSR, minus fruit, nut and seed species**			
Multi-adjusted model - HR (95% CI)[a]	0·89 (0·88-0·90)	<0·001	-1%
**DSR, minus dairy species**			
Multi-adjusted model - HR (95% CI)[a]	0·91 (0·90-0·91)	<0·001	1%
**DSR, minus cereal species**			
Multi-adjusted model - HR (95% CI)[a]	0·91 (0·90-0·92)	<0·001	1%
**DSR, minus meat species**			
Multi-adjusted model - HR (95% CI)[a]	0·90 (0·89-0·91)	<0·001	0%
**DSR, minus fish and shellfish species**			
Multi-adjusted model - HR (95% CI)[a]	0·89 (0·88-0·90)	<0·001	-1%
**DSR, minus egg species**			
Multi-adjusted model - HR (95% CI)[a]	0·91 (0·90-0·92)	<0·001	1%
**DSR, minus condiment species**			
Multi-adjusted model - HR (95% CI)[a]	0·90 (0·90-0·91)	<0·001	0%

[a]Multi-adjusted models were stratified for centre, age at recruitment (1-y intervals, time-scale), and sex and adjusted for baseline alcohol intake (g/day), physical activity (Cambridge index: active; moderately active; moderately inactive; inactive; missing), marital status (single, divorced, separated, or widowed; married or living together; unknown), smoking status and intensity of smoking (current, 1-15 cigarettes/day; current, 16-25 cigarettes/day; current, 26+ cigarettes/day; current, pipe/cigar/occasional; current/former, missing; former, quit 11-20y; former, quit 20+y; former, quit ≤ 10y; never; unknown), educational level [longer education (incl. university degree, technical or professional school); secondary school; primary school completed; not specified], baseline energy intake (kcal/day), baseline fibre intake (g/day), baseline red and processed meat consumption (g/day), and an 18-point Mediterranean diet score [49].

Abbreviations: CI, confidence interval; DSR, dietary species richness; HR, hazard ratio.
