# Supplementary material for: Food biodiversity and total and cause-specific mortality in 9 European countries: An analysis of a prospective cohort study
Source: PLoS Med. 2021 Oct 18;18(10):e1003834. doi: 10.1371/journal.pmed.1003834 (PMC8559947; doi:10.1371/journal.pmed.1003834)
Supplement: S6 Table — CI, confidence interval; EPIC, European Prospective Investigation into Cancer and Nutrition; HR, hazard ratio. (PDF) [file pmed.1003834.s009.pdf]

S6 Table. E-values for HR and 95% CI, associations between dietary species richness and total and cause-specific mortality rates from multivariable Cox proportional hazards regression models, EPIC cohort, 1992-2014. EPIC, European Prospective Investigation into Cancer and Nutrition.

	Per 10-species increment		Q_1	Q_2		Q_3		Q_4		Q_5	
	HR (95% CI)	E-value	HR (95% CI)	HR (95% CI)	E-value	HR (95% CI)	E-value	HR (95% CI)	E-value	HR (95% CI)	E-value
**Total mortality**	0·90 (0·89-0·90)	1·46 (1·46)	1·00 (ref)	0·91 (0·88-0·94)	1·43 (1·32)	0·80 (0·76-0·83)	1·81 (1·70)	0·69 (0·66-0·72)	2·26 (2·12)	0·63 (0·59-0·66)	2·55 (2·40)
**Cancer**	0·93 (0·92-0·95)	1·36 (1·29)	1·00 (ref)	0·92 (0·87-0·97)	1·39 (1·21)	0·87 (0·82-0·93)	1·56 (1·36)	0·78 (0·73-0·83)	1·88 (1·70)	0·75 (0·69-0·82)	2·00 (1·74)
**CVD**	0·88 (0·85-0·91)	1·53 (1·43)	1·00 (ref)	0·94 (0·86-1·03)	1·32 (1·00)	0·78 (0·69-0·87)	1·88 (1·56)	0·65 (0·57-0·74)	2·45 (2·04)	0·56 (0·49-0·65)	2·97 (2·45)
**CHD**	0·87 (0·84-0·90)	1·56 (1·46)	1·00 (ref)	0·89 (0·80-0·99)	1·50 (1·11)	0·75 (0·64-0·86)	2·00 (1·60)	0·67 (0·58-0·78)	2·35 (1·88)	0·55 (0·46-0·65)	3·04 (2·45)
**Respiratory disease**	0·84 (0·80-0·88)	1·67 (1·53)	1·00 (ref)	0·93 (0·80-1·09)	1·36 (1·00)	0·75 (0·62-0·91)	2·00 (1·43)	0·56 (0·46-0·69)	2·97 (2·26)	0·44 (0·34-0·55)	3·97 (3·04)
**Digestive disease**	0·80 (0·76-0·86)	1·81 (1·60)	1·00 (ref)	0·98 (0·80-1·21)	1·16 (1·00)	0·64 (0·49-0·83)	2·50 (1·70)	0·53 (0·41-0·69)	3·18 (2·26)	0·46 (0·34-0·63)	3·77 (2·55)

Multi-adjusted models were stratified for centre, age at recruitment (1-y intervals, time-scale), and sex and adjusted for baseline alcohol intake (g/day), physical activity (Cambridge index: active; moderately active; moderately inactive; inactive; missing), marital status (single, divorced, separated, or widowed; married or living together; unknown), smoking status and intensity of smoking (current, 1-15 cigarettes/day; current, 16-25 cigarettes/day; current, 26+ cigarettes/day; current, pipe/cigar/occasional; current/former, missing; former, quit 11-20y; former, quit 20+y; former, quit ≤ 10y; never; unknown), educational level [longer education (incl. university degree, technical or professional school); secondary school; primary school completed; not specified], baseline energy intake (kcal/day), baseline fibre intake (g/day), baseline red and processed meat consumption (g/day), and an 18-point Mediterranean diet score [1]. E-values were calculated from VanderWeele *et al*. [53] and Mathur *et al.* [54].
Abbreviations: CHD, coronary heart disease; CI, confidence interval; CVD, cardiovascular disease; HR, hazard ratio.
