# Supplementary material for: Food biodiversity and total and cause-specific mortality in 9 European countries: An analysis of a prospective cohort study
Source: PLoS Med. 2021 Oct 18;18(10):e1003834. doi: 10.1371/journal.pmed.1003834 (PMC8559947; doi:10.1371/journal.pmed.1003834)
Supplement: S1 Text — EPIC, European Prospective Investigation into Cancer and Nutrition. (PDF) [file pmed.1003834.s011.pdf]

S1 Text. Analysis plan extracted from the project proposal submitted to and approved (March, 2019) by the EPIC Steering Committee. EPIC, European Prospective Investigation into Cancer and Nutrition.

PROJECT DESCRIPTION

**Main Research Question**
The present study will assess how food biodiversity in diets is associated with all-cause and cause-specific mortality in Europe. It will estimate the potential health benefits of promoting speciesrichness, evenness, or dissimilarity in diets in Europe and contribute to the development of recommendations that include variety of species in food based dietary guidelines.
**Specific Aims**
Primary exposure<br>To assess associations of food biodiversity with all-cause and cause-specific mortality. Quantiles of usual dietary species richness (# species/year) will be used as primary exposure of food biodiversity in diets, using the available FFQ data. Species richness will be considered for the total diet as well as for each individual food group.

[…]

**Design and Analysis Plan**
All foods (and drinks) reported in the dietary questionnaires will be attributed to the corresponding species - where possible - with a unique species identifier. Taxonomic reference will be used for disambiguation. As small quantities of foods consumed are important from a biodiversity point of view, no minimum quantity will be set. However, sensitivity analyses will be run using different minimum quantity cut-offs.<br><br>A matrix with all foods and recipes consumed in the EPIC cohort was prepared and allows recoding of foods independent from the personal data in the EPIC study at Ghent University. Final matching ofspecies consumed with personal data in the EPIC database will take place at IARC.<br><br>Total number of species usually consumed (in the total diet and per food group) will be used as primary exposure of food biodiversity in diets and included in the models as a continuous variable as well as in categories using quantiles.<br><br>The primary end point of the present analysis is mortality from any cause. Secondary outcomes are cause-specific mortality rates known to be associated with poor diets.<br><br>Data will be analysed using SAS, version 9.4. Multivariable Cox proportional hazards models will be fitted to compute hazard ratios and 95% confidence intervals. Models will be adjusted for study centre, sex, age of the participant at recruitment, and total energy intake, and potential confounding factors using the 5% CIE criterion. All statistical tests will be two-sided and $P<0.05$ is considered as statistically significant. *P*-values for trend will be calculated across the categories of food biodiversity diets with the use of the Wald test.
